# Identification and validation of immunotherapy for four novel clusters of colorectal cancer based on the tumor microenvironment

**DOI:** 10.3389/fimmu.2022.984480

**Published:** 2022-10-28

**Authors:** Xiaoyong Zheng, Yajie Ma, Yan Bai, Tao Huang, Xuefeng Lv, Jinhai Deng, Zhongquan Wang, Wenping Lian, Yalin Tong, Xinyu Zhang, Miaomiao Yue, Yan Zhang, Lifeng Li, Mengle Peng

**Affiliations:** ^1^ Department of Digestion, Henan Provincial Third People’s Hospital, Zhengzhou, China; ^2^ Department of Medical Affair, Henan Provincial Third People’s Hospital, Zhengzhou, China; ^3^ Department of Digestion, Zhengzhou First People’s Hospital, Zhengzhou, China; ^4^ Medical School, Huanghe Science and Technology University, Zhengzhou, China; ^5^ Department of Clinical Laboratory, The First Affiliated Hospital of Zhengzhou University, Zhengzhou, China; ^6^ Richard Dimbleby Department of Cancer Research, Comprehensive Cancer Centre, Kings College London, London, United Kingdom; ^7^ Department of Clinical Laboratory, Henan Provincial Third People’s Hospital, Zhengzhou, China; ^8^ Department of Digestion, The First Affiliated Hospital of Zhengzhou University, Zhengzhou, China; ^9^ Cancer Center, The First Affiliated Hospital of Zhengzhou University, Zhengzhou, China; ^10^ Internet Medical and System Applications of National Engineering Laboratory, Zhengzhou, China

**Keywords:** colorectal cancer, tumor microenvironment, clusters, immunotherapy, bioinformatics

## Abstract

The incidence and mortality of colorectal cancer (CRC) are increasing year by year. The accurate classification of CRC can realize the purpose of personalized and precise treatment for patients. The tumor microenvironment (TME) plays an important role in the malignant progression and immunotherapy of CRC. An in-depth understanding of the clusters based on the TME is of great significance for the discovery of new therapeutic targets for CRC. We extracted data on CRC, including gene expression profile, DNA methylation array, somatic mutations, clinicopathological information, and copy number variation (CNV), from The Cancer Genome Atlas (TCGA), Gene Expression Omnibus (GEO) (four datasets—GSE14333, GSE17538, GSE38832, and GSE39582), cBioPortal, and FireBrowse. The MCPcounter was utilized to quantify the abundance of 10 TME cells for CRC samples. Cluster repetitive analysis was based on the Hcluster function of the Pheatmap package in R. The ESTIMATE package was applied to compute immune and stromal scores for CRC patients. PCA analysis was used to remove batch effects among different datasets and transform genome-wide DNA methylation profiling into methylation of tumor-infiltrating lymphocyte (MeTIL). We evaluated the mutation differences of the clusters using MOVICS, DeconstructSigs, and GISTIC packages. As for therapy, TIDE and SubMap analyses were carried out to forecast the immunotherapy response of the clusters, and chemotherapeutic sensibility was estimated based on the pRRophetic package. All results were verified in the TCGA and GEO data. Four immune clusters (ImmClust-CS1, ImmClust-CS2, ImmClust-CS3, and ImmClust-CS4) were identified for CRC. The four ImmClusts exhibited distinct TME compositions, cancer-associated fibroblasts (CAFs), functional orientation, and immune checkpoints. The highest immune, stromal, and MeTIL scores were observed in CS2, in contrast to the lowest scores in CS4. CS1 may respond to immunotherapy, while CS2 may respond to immunotherapy after anti-CAFs. Among the four ImmClusts, the top 15 markers with the highest mutation frequency were acquired, and CS1 had significantly lower CNA on the focal level than other subtypes. In addition, CS1 and CS2 patients had more stable chromosomes than CS3 and CS4. The most sensitive chemotherapeutic agents in these four ImmClusts were also found. IHC results revealed that CD29 stained significantly darker in the cancer samples, indicating that their CD29 was highly expressed in colon cancer. This work revealed the novel clusters based on TME for CRC, which would guide in predicting the prognosis, biological features, and appropriate treatment for patients with CRC.

## Introduction

Colorectal cancer (CRC) is a common malignant tumor in the digestive system. In recent years, the incidence of CRC is gradually increasing, and the mortality is also on the rise, ranking at the forefront of all malignant tumors, seriously endangering human health (1). The treatment of CRC is based on radical surgery, supplemented by chemotherapy, but nearly half of the patients are still trapped in tumor recurrence or metastasis without effective treatment (2). The traditional clinical and pathological predictors of CRC mainly include intestinal obstruction, pathological stage, level of cell differentiation, invaded vessels, invaded nerves, microsatellite status, etc. However, the final clinical significance is not very obvious. Recently, in order to more accurately predict the prognosis of patients with CRC, more and more researchers have begun to pay attention not only to tumor cells themselves but also to the tumor microenvironment (TME) of tumor cells.

The occurrence of CRC is a multistage mutation accumulation process involving multiple oncogenes, and the TME also plays an important role in the regulation (3) and drug resistance (4, 5) of CRC. The TME consists of a variety of cell types, including immune cells, inflammatory cells, adipocytes, fibroblasts, and vascular endothelial cells, as well as non-cellular components in and around the tumor (6). TME cells can be induced by tumor cells to produce a large number of cytokines and growth factors, thus forming a microenvironment conducive to the survival and proliferation of tumor cells. The TME can mediate the immune escape of tumor cells with the participation of tumor-associated immunosuppressive molecules (transforming growth factor-β, TGF-β), tumor-associated immunosuppressive cells (tumor-associated macrophages, TAMs), and tumor-associated proinflammatory responses (tumor-associated neutrophils) (7). The cellular components in the TME have become key modulators of tumor progression, organ-specific metastasis, and therapeutic response, among which tumor-infiltrating immune cells are the key to immunotherapy (8). Furthermore, tumor-infiltrating lymphocytes (TILs) can directly affect the prognosis and response to immunotherapy (9).

The heterogeneity of tumors is one of the important characteristics of tumors, which enables tumors to evolve various characteristics to adapt to the environment and even to resist the treatment of tumors (10, 11). Traditionally, tumors have been classified according to the type of cell or tissue they originate from and, thus, have a “one-size-fits-all” approach to pathology and treatment. It was not until sequencing became widely available that we realized that there were differences in genomic, transcriptome, and epigenetic characteristics within the same type of tumor (10). For example, the CRC Subtyping Consortium proposed a consensus molecular model, which divided CRC into four consensus molecular subtypes (CMS) according to pathological features (12). CMS1 is the type involved in microsatellite instability (MSI), also known as a high mutation type, which is manifested by mismatched gene repair changes. CMS2 is typical and is associated with abnormal activation of WNT or MYC signaling pathways. CMS3 is a metabolic type, showing a high mutation degree of KRAS and metabolic disorder. CMS4 indicates an abnormal activation of the TGF-β signaling pathway. Even within the same tumor, its genetic characteristics are different between subcellular populations and change dynamically as the tumor develops (11, 13). The understanding of tumor heterogeneity has led to a more detailed classification of tumors, and the development of different treatment regimens based on the molecular characteristics of tumors has improved the therapeutic outcomes of multiple tumors. For example, imatinib is used to treat BCR-ABL tyrosine kinase constitutively activated chronic myeloid leukemia (14), HER2 protein-targeting drugs are used to treat HER2-positive breast cancer (15), and estrogen antagonists are used to treat estrogen receptor-positive breast cancer (16).

In this study, we integrated TME cells of CRC to identify four immune clusters (ImmClust-CS1, ImmClust-CS2, ImmClust-CS3, and ImmClust-CS4), which were validated using data from the Gene Expression Omnibus (GEO) datasets. We described each according to their biological characteristics, including the prognosis, immune status, somatic mutations, copy number variation (CNV), and response to treatment.

## Materials and methods

### Public data acquisition and preprocessing

The RNA-seq FPKM (fragments per kilobase million) data of TCGA-COAD and TCGA-READ were downloaded from the UCSC Xena platform (https://xenabrowser.net/) (17). After primary tumor selection, a total of 390 COAD and 154 READ samples were included in our study. The FPKM style of RNA-seq data was normalized into TPM value (18). The 450K DNA methylation array was also extracted from the UCSC Xena platform (19). The somatic mutation data and the clinicopathological information of patients with COAD or READ were obtained from the cBioPortal platform (http://www.cbioportal.org/datasets) (20). The data on CNV were acquired from FireBrowse (http://firebrowse.org/) (21). Four external independent datasets, namely, GSE14333, GSE17538, GSE38832, and GSE39582, were downloaded from the GEO database and quantitated by Affymetrix Human Genome U133 Plus 2.0 Array (22–25). TCGA and GEO data were combined to remove batch effects by ComBat in R package SVA (26), and the removal of batch effects was tested by principal component analysis (PCA) (27).

### TME abundance quantification and immune cluster establishment

MCPcounter is an R package that quantifies the absolute abundance of eight immune cells (B-cell lineage, CD8^+^ T cells, cytotoxic lymphocytes, monocytic lineage, myeloid dendritic cells, natural killer cells, neutrophils, and T cells) and two stromal cells (fibroblasts and endothelial cells) using transcriptome data (28). We utilized MCPcounter to quantify the abundance of the 10 TME cells for CRC samples. After cluster repetitive analysis based on Hcluster function of Pheatmap package in R (29), four immune clusters (ImmClust-CS1, ImmClust-CS2, ImmClust-CS3, and ImmClust-CS4) were identified. The ESTIMATE R package was applied to compute immune scores and stromal scores (30), representing the enrichment scores for CRC patients.

### Immunotherapy response analysis

Based on the ESTIMATE tool, the present study used gene expression data from CRC to estimate stromal and immune cells in cancer tissue to predict the immune score and stromal score in CRC (30). TILs were associated with the clinical outcomes of CRC (31, 32). To further evaluate the local tumor immune response of the four ImmClusts, genome-wide DNA methylation profiling was applied and transformed into methylation of tumor-infiltrating lymphocyte (MeTIL) using PCA analysis (33). TIDE is a computational method for predicting immune checkpoint blockade (ICB) responses (34). Based on RNA expression profiles, TIDE prediction scores were calculated to forecast the likelihood of CRC patients responding to immunotherapy. A lower TIDE score indicated a lower possibility of immune escape (34). In addition, SubMap analysis was carried out to contrast gene expression similarity between ImmClusts and the responders of anti-PD-1 or anti-CTLA-4 therapy (35–38).

### Assessment of cancer-associated fibroblasts

Cancer-associated fibroblasts were reported to play an essential role in the TME of CRC (38). Since cancer-associated fibroblast (CAF) may have modeled different patient subpopulations, CAF-related genes and signatures were mapped to ImmClusts. Previous studies provided seven CAF-related genes, namely, *ACTA2*, *PDGFRA*, *PDGFRB*, *THY1*, *COL1A1*, *FAP*, and *PDPN* (39–43). We obtained eight CAF-related signatures (ecm-myCAF, detox-iCAF, IL-iCAF, TGFB-myCAF, wound-myCAF, IFNG-iCAF, CAF-S1, and normal fibroblast) from the study based on single-cell analysis (44).

### Evaluation of mutation differences

The MOVICS package is designed for multi-omics comprehensive clustering and visualization of cancer clusters, which provides a unified interface and standardizes the output for 10 algorithms (CIMLR, iClusterBayes, MoCluster, COCA, ConsensusClustering, IntNMF, LRAcluster, NEMO, PINSPlus, and SNF) (45). The ImmClusts of the TCGA-CRC cohorts were comprehensively characterized by the MOVICS package, including somatic mutation (46), tumor mutational burden (TMB), and fraction genome altered score. The package DeconstructSigs can put 96 mutation spectrums into 30 corresponding mutation signatures of the COSMIC database (47). Mutations mediated by the apolipoprotein B mRNA-editing enzyme catalytic polypeptide-like (APOBEC) family are widespread in human cancers (48). APOBEC has been reported to be associated with immunotherapy response (49). We selected two APOBEC-related signatures and weighted them to obtain the weight of the APOBEC mutant signature. Somatic CNV (SCNA) may affect as many as thousands of genes simultaneously, but the selective advantage that drives variation may be mediated by only one or a few of these genes. Based on the Genomic Identification of Significant Targets in Cancer (GISTIC) algorithm (50), we compared the chromosomal instability of subtypes.

### Prediction of the sensibility of chemotherapeutics

A wide range of drug screening can be performed through the Genomics of Drug Sensitivity in Cancer (GDSC) website (51). Based on pRRophetic package in R (52, 53), Ridge’s regression was used to construct a prediction model between drug sensitivity and the expression profile of cell lines. Whereafter, we applied the aforementioned model to estimate the half-maximal inhibitory concentration (IC_50_) value of chemotherapeutics for CRC patients.

### Human tissue sample collection

Formalin-fixed and paraffin-embedded (PPFE) specimens were collected from cancerous and paracancerous tissues of CRC patients in the First Affiliated Hospital of Zhengzhou University. All the samples were stored at room temperature (20°C–25°C). According to pathological features, at least two pathologists diagnosed all the specimens and reached an agreement. Lastly, this study included 10 cases of CRC cancerous and paracancerous tissues. The study was approved by the Ethics Committee of the First Affiliated Hospital of Zhengzhou University (Ethics No. 2021-KY-0147-002).

### Immunohistochemical staining

According to the manufacturers’ protocol, immunohistochemistry (IHC) staining of CRC cancerous and paracancerous tissues was performed. First, we dewaxed, hydrated, and blocked the paraffin-embedded sections and then incubated them overnight at 4°C with a CD29 antibody (Affinity, China). The next day, sections were washed three times with PBS and then incubated with secondary antibodies at 37°C. Again, sections were washed with PBS, dropped into a DAB reagent, and restained with hematoxylin for 2 min. Finally, these sections were visualized by light microscopy, and the results of Masson staining and IHC were analyzed using ImageJ software.

## Results

### Immune-related cluster establishment

**Figure 1:**
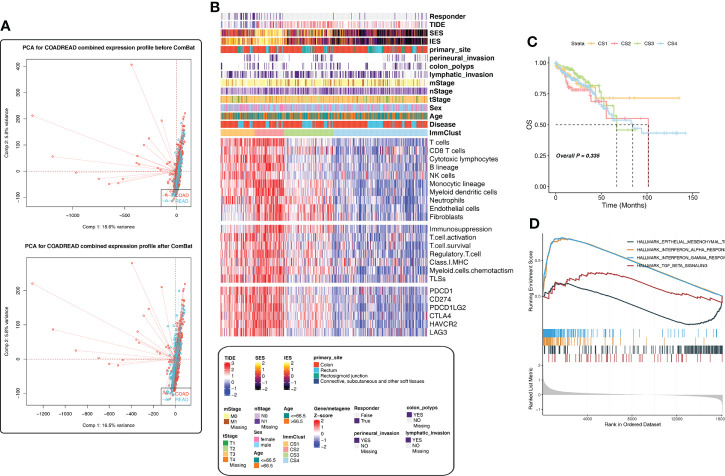
Immune-related cluster establishment for The Cancer Genome Atlas (TCGA). **(A)** Batch effect removal of the TCGA-COAD and TCGA-READ cohorts using PCA analysis. **(B)** The heatmap showed the differences in the distribution of the four ImmClusts in clinicopathological features, TME compositions, functional orientation, immune checkpoints, and so on. **(C)** K‐M survival curves showed the differences of overall survival and recurrence rate among the four ImmClusts. **(D)** GSEA analysis comparing the differences of enrichment pathways between CS1 and the other ImmClusts.

If the sample dataset collection time, collection institutions, sequencing platform, and other factors are different, they may automatically form different batches, thus affecting the real data. Therefore, batch effects should be checked and removed before subsequent analysis; otherwise, all subsequent analysis results will be invalid. **Figure 1A** shows the PCA diagram of the TCGA-COAD and TCGA-READ data before and after batch removal, indicating that batch effect removal was relatively successful.

We developed an immune-related cluster using the Hcluster function of the Pheatmap package, and four immune clusters (ImmClust-CS1, ImmClust-CS2, ImmClust-CS3, and ImmClust-CS4) were identified for CRC (**Figure 1B**). The heatmap showed the differences in the distribution of the four ImmClusts in clinicopathological features, TME compositions, functional orientation, and immune checkpoints (**Figure 1B**). The four ImmClusts exhibited distinct TME compositions. ImmClust-CS1 was characterized by a high enrichment of immune cells and low fibroblasts (**Figure 1B**). ImmClust-CS2 was dominated by immune-cell-related genes, as well as endothelial cells and fibroblasts (**Figure 1B**). ImmClust-CS3 and ImmClust-CS4 were both characterized by immune low and fibroblast high and fibroblast low, respectively (**Figure 1B**). As for the functional orientation (immunosuppression, T-cell activation, T-cell survival, regulatory T cells, major histocompatibility complex class I, myeloid cell chemotaxis, and tertiary lymphoid structures), on the whole, the expression values of related genes were relatively high in ImmClust-CS1 and ImmClust-CS2 and relatively low in ImmClust-CS3 and ImmClust-CS4 (**Figure 1B**). The expression of immune checkpoint genes was consistent with the above findings (**Figure 1B**).

Furthermore, we compared the clinical outcomes of patients in the four ImmClusts. Although the survival patterns of the four ImmClusts exhibited were not statistically significant (*P* = 0.335), ImmClust-CS1 did not reach the median survival period in 10 years and had a better prognosis than other ImmClusts (**Figure 1C**). Hence, we compared the differences in enrichment pathways between CS1 and the other ImmClusts. GSEA analysis showed that compared with the other three ImmClusts, CS1 was enriched in interferon-alpha response, interferon-gamma response, and TGF beta signaling pathways, while the epithelial–mesenchymal transition (EMT) pathway was downregulated, indicating that CS1 was enriched in immune-related pathways but downregulated in the EMT pathway (**Figure 1D**).

### Immunotherapy response analysis

**Figure 2:**
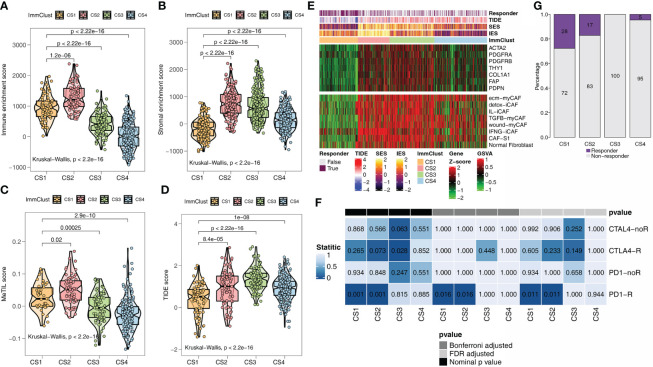
Immunotherapy response analysis for the TCGA. **(A)** Immune enrichment score for the four ImmClusts. **(B)** Stromal enrichment score for the four ImmClusts. **(C)** MeTIL score for the four ImmClusts. **(D)** TIDE score for the four ImmClusts. **(E)** Response to immunotherapy of the four ImmClusts. **(F)** The heatmap showed the differences in the distribution of the four ImmClusts in cancer-associated fibroblast (CAF)-related genes and signatures. **(G)** SubMap analysis for the four ImmClusts.

ESTIMATE is an algorithm that uses transcription profiles of cancer samples to estimate the number of tumor cells, as well as the number of infiltrated immune and stromal cells. Among the ESTIMATE scores of the four ImmClusts, the highest immune (**Figure 2A**) and stromal scores (**Figure 2B**) were observed in CS2, in contrast to the lowest scores in CS4 (**Figures 2A, B** **)**. A study has shown that the MeTIL score system may assess immune and immunotherapy responses in CRC (54). Interestingly, our data suggested that CS2 had the highest MeTIL score (**Figure 2C**), while CS4 had the lowest MeTIL score, which further suggested the differences in immunotherapy responses among the four ImmClusts. A lower TIDE score indicated a lower possibility of immune escape. From **Figures 2D, E**, we found that patients in CS1 were more likely to respond to immunotherapy (Fisher’s exact test, *P* < 0.001). In mapping CAF-related genes and signatures to ImmClusts, we found a highly positive correlation between CS2 and CAFs (**Figure 2F**). At the same time, the immune gene expression profiles of CS1 and CS2 were found to be similar to those of melanoma patients responding to anti-PD-1 therapy using SubMap analysis (**Figure 2G**). To sum up, CS1 may respond to immunotherapy, while CS2 may respond to immunotherapy after anti-CAFs.

### Verification analysis based on GEO data

**Figure 3:**
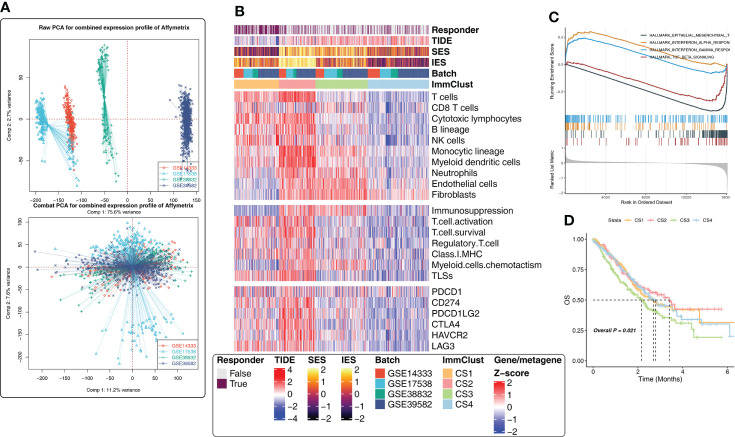
Immune-related cluster establishment for the Gene Expression Omnibus (GEO). **(A)** Batch effect removal of four external independent datasets using PCA analysis. **(B)** The heatmap showed the differences in the distribution of the four ImmClusts in clinicopathological features, TME compositions, functional orientation, immune checkpoints, and so on. **(C)** GSEA analysis comparing the differences of enrichment pathways between CS1 and the other ImmClusts. **(D)** K–M survival curves showed the differences of overall survival and recurrence rate among the four ImmClusts.

To validate the results of the above analysis, four external CRC cohorts from the GEO database were included in the follow-up study. First, the data of the GEO cohorts were combined and PCA analysis was performed. The batch effect removal was observed successfully (**Figure 3A**). Subsequently, considering the large cohort size and uneven distribution of tumor purity in GEO, we removed the samples with a tumor purity larger than 0.8 (the higher the tumor purity, the less accurate the TME estimate), leaving 833 CRC samples. Here, four subtypes were obtained by unsupervised clustering, and the distribution of subtypes in clinicopathological features, TME compositions, functional orientation, and immune checkpoints was consistent with the TCGA cohort (**Figure 3B**). Happily, for survival analysis, we found statistically significant differences in survival curves, and CS1 and CS2 subgroups with high levels of immune cell infiltration fared better, while CS3 with lower immune cell infiltration but higher fibroblast infiltration had a poor prognosis (**Figure 3D**). Conclusively, GSEA analysis showed that CS1 was associated with interferon-alpha response, interferon-gamma response, TGF beta signaling, and epithelial–mesenchymal transition pathways (**Figure 3C**), which was consistent with the previous findings.

**Figure 4:**
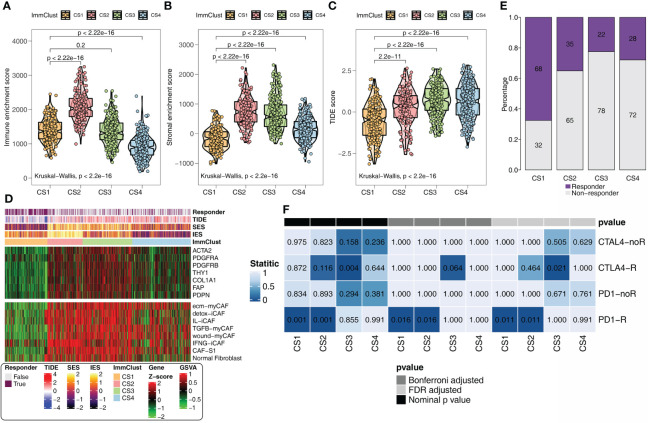
Immunotherapy response analysis for the GEO. **(A)** Immune enrichment score for the four ImmClusts. **(B)** Stromal enrichment score for the four ImmClusts. **(C)** TIDE score for the four ImmClusts. **(D)** Response to immunotherapy of the four ImmClusts. **(E)** SubMap analysis for the four ImmClusts. **(F)** The heatmap showed the differences in the distribution of the four ImmClusts in CAF-related genes and signatures.

In addition, the same results as the above findings were confirmed. The immune (**Figure 4A**) and stromal (**Figure 4B**) scores were the highest in CS2 and the lowest in CS4. CS1 with the lowest TIDE score (**Figure 4C**) remained the subgroup most likely to respond to immunotherapy (**Figure 4D**). The heatmap revealed that CS2 was positively correlated with CAF-related genes and signatures (**Figure 4F**). SubMap analysis uncovered that CS1 and anti-CAF-CS2 may respond to anti-PD-1 immunotherapy (**Figure 4E**).

### Evaluation of mutation differences

**Figure 5:**
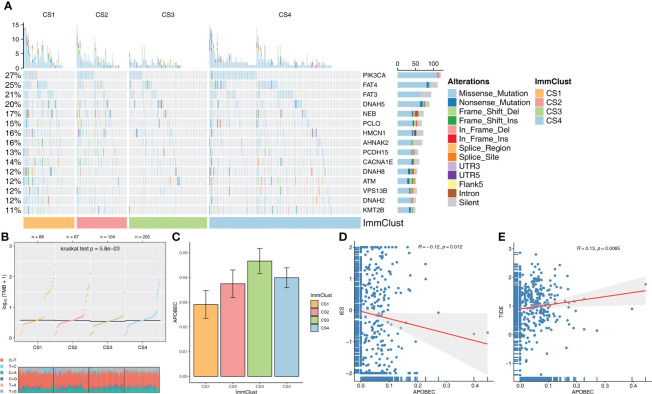
Evaluation of mutation differences among the four ImmClusts. **(A)** The waterfall plot of somatic mutation features established with ImmClusts. **(B)** TMB and distribution of TiTv calculated by MOVICS for the four ImmClusts. **(C)** Apolipoprotein B mRNA-editing enzyme catalytic polypeptide-like (APOBEC) mutation weights of the four ImmClusts. **(D)** Correlation between immune enrichment score (IES) and APOBEC mutation weight. **(E)** Correlation between TIDE score and APOBEC mutation weight.

The distribution variations of the somatic mutations among the four ImmClusts were also analyzed based on MOVICS. The top 15 markers with the highest mutation frequency were PIK3CA, FAT4, FAT3, DNAH5, NEB, PCLO, HMCN1, AHNAK2, PCDH15, CACNA1E, DNAH8, ATM, VPS13B, DNAH2, and KMT2B (**Figure 5A**). TMB and TiTv were calculated by MOVICS, and it was found that CS1 had a higher TMB (**Figure 5B**). As for APOBEC, mutation weights were significantly different among the four ImmClusts, with CS3 having the highest and CS1 the lowest (**Figure 5C**). In addition, we found a significant negative correlation between immune enrichment score (IES) and APOBEC mutation weight (*R* = −0.12, *P* = 0.012, **Figure 5D**), while APOBEC mutation weight was positively correlated with TIDE score (*R* = 0.13, *P* = 0.0065, **Figure 5E**), suggesting that APOBEC is involved in immunotherapy response.

**Figure 6:**
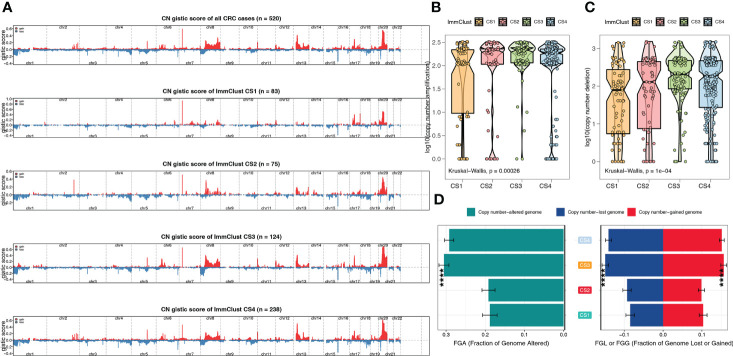
The chromosomal instability of the four ImmClusts. **(A)** Manhattan plot showing the CNV at the chromosomal level. **(B)** Copy number amplification of the four ImmClusts. **(C)** Copy number deletion of the four ImmClusts. **(D)** Fraction genome-altered scores of the four ImmClusts. ****p < 0.001.

From the Manhattan plot, we can see CNV at the chromosomal level, which was computed by the GISTIC algorithm (**Figure 6A**). By counting copy number amplification (**Figure 6B**) and deletion (**Figure 6C**), respectively, we found that CS1 had a significantly lower CNA at the focal level than other subtypes. Fraction genome-altered scores (threshold 0.2) were calculated using MOVICS packages to characterize chromosomal instability. The results showed that CS1 and CS2 patients had more stable chromosomes than CS3 and CS4 (**Figure 6D**).

### Univariate Cox regression analysis for CAF-related genes

**Figure 7:**
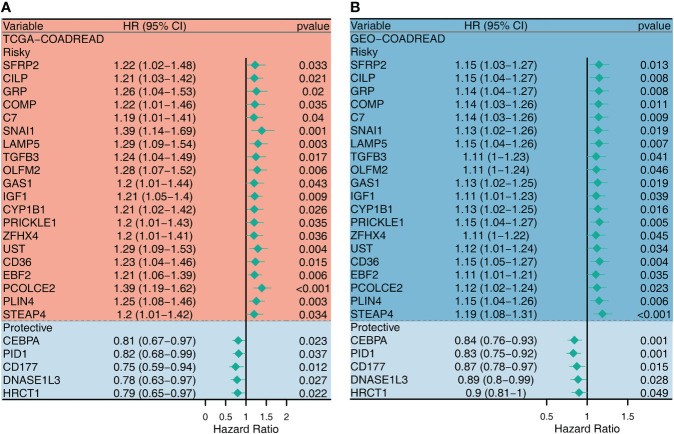
Univariate Cox regression analysis for the CAF-related genes in TCGA **(A)** and GEO **(B)** datasets.

Since CAF plays an important role in shaping the ImmClusts of CRC, 289 CAF-related genes (CRGs) were extracted from the literature and analyzed by univariate Cox regression analysis. In the TCGA-CRC cohort, 49 risky CRGs and 8 protective CRGs were identified. In the GEO-CRC dataset, 56 risky CRGs and 13 protective CRGs were found. We selected 25 intersection genes to map the forest plot. As can be seen from **Figure 7**, there were 20 risky CRGs (*SERP2*, *CILP*, *GRP*, *COMP*, *C7*, *SNAI1*, *LAMP5*, *TGFB3*, *OLFM2*, *GAS1*, *IGF1*, *CYP1B1*, *PRICKLE1*, *ZFHX4*, *UST*, *CD36*, *EBF2*, *PCOLCE2*, *PLIN4*, and *STEAP4*) and 5 protective CRGs (*CEBPA*, *PID1*, *CD177*, *DNASE1L3*, *HRCT1*) in the intersection genes (**Figure 7**).

### Prediction of the sensibility of chemotherapeutics

**Figure 8:**
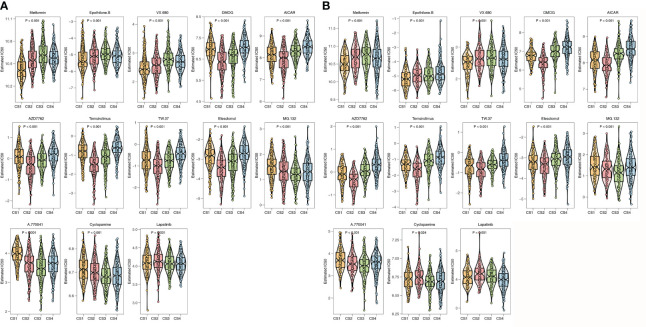
Prediction of chemotherapeutic sensibility. **(A)** Prediction of chemotherapeutic sensibility for the TCGA. **(B)** Prediction of chemotherapeutic sensibility for the GEO.

To further investigate the treatment strategies for the four ImmClusts of CRC, we conducted a prediction of the sensibility of chemotherapeutics to evaluate the IC_50_ value using the pRRophetic package. The IC_50_ value can be used to measure the ability of a drug to induce apoptosis, that is, the higher the inducing ability, the lower the value, and of course, it can also be used to reverse the tolerance of a certain cell to the drug. We screened out drugs that showed consistent sensitivity in the TCGA (**Figure 8A**) and GEO (**Figure 8B**) databases for display. Compared with the other three ImmClusts, patients in CS1 were most sensitive to metformin, epothilone B, and VX-680; patients in CS2 were most sensitive to DMOG, AICAR, AZD7762, temsirolimus, TW.37, and elesclomol; patients in CS3 were most sensitive to MG.132, A.770041, and cyclopamine; and patients in CS4 were most sensitive to lapatinib.

### Validation of the protein expression levels of CD29 using IHC

**Figure 9:**
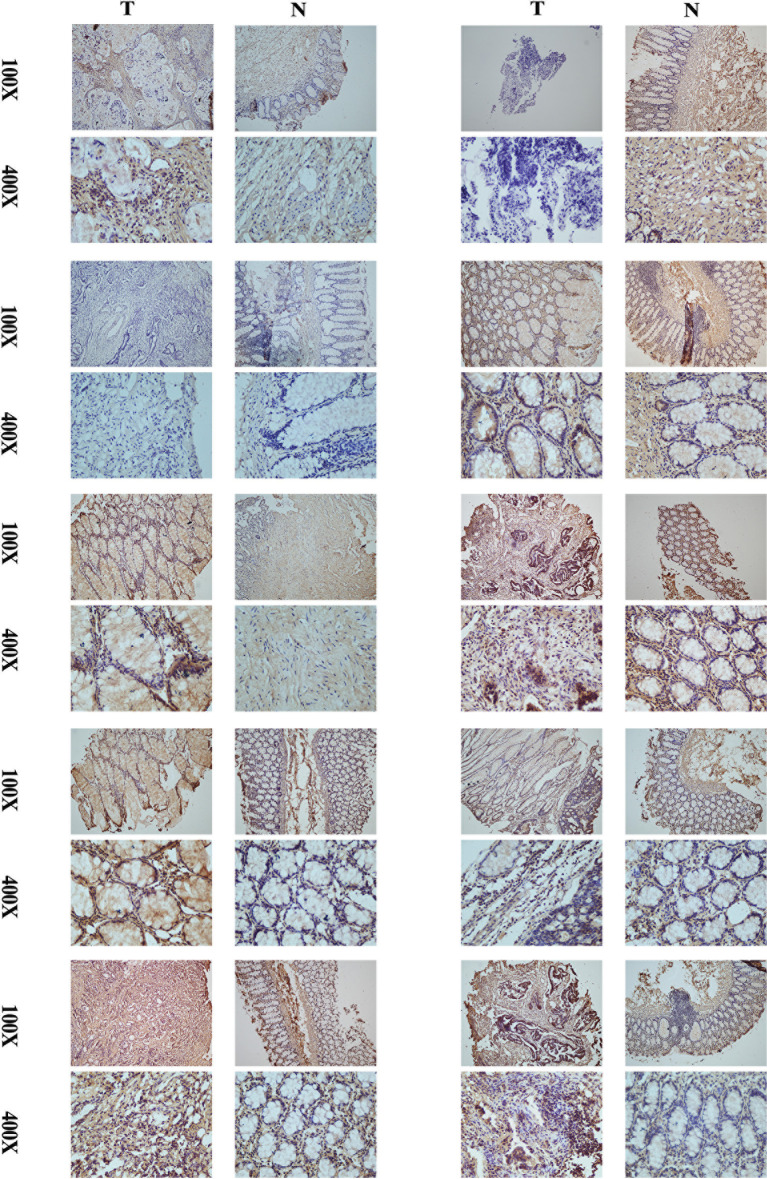
Comparison of cancer and paracancer IHC results revealed that CD29 stained significantly darker in the cancer samples, indicating that their CD29 was highly expressed in colon cancer.

A comparison of cancer and paracancer IHC results revealed that CD29 stained significantly darker in the cancer samples, indicating that CD29 was highly expressed in colon cancer (**Figure 9**). These results are consistent with our subtype results, indicating the reliability and reproducibility of the classification.

## Discussion

CRC is the third most common malignant tumor in the world and the second leading cause of cancer death worldwide, with about 1.8 million new cases and 800,000 deaths every year (55, 56). The tumor microenvironment plays an important role in tumor genesis, development, and tumor immunity. Studies have shown that the infiltration and metastasis of immune cells, memory CD4^+^ T cells, and CD8^+^ T cells in the TME can regulate tumor immunity and participate in the three stages of tumor cell clearance, tumor and body balance, and tumor immune escape (57). They not only inhibit tumor growth but also screen tumors for hypoimmunogenicity, leading to tumor escape (58).

Our data revealed that CS1 was enriched in the interferon-alpha (IFN-α) response and interferon-gamma (IFN-γ) response signaling pathways, while it was downregulated in the EMT pathway. EMT is the process by which cells lose epithelial features and gain mesenchymal properties, such as increased motility of tumor cells (59). EMT processes are a series of changes and transformations resulting from environmental stimuli, depending on the organization and signal transduction environment (60). In CRC, EMT is strongly associated with aggressive or metastatic phenotypes (61). In-depth research on the mechanism of action and interaction of the related signaling pathways in CRC EMT will help to develop more new methods for CRC treatment and promote individualized treatment. IFN-α can inhibit the proliferation of tumor cells through the adaptive and innate immune system (62). IFN-α can also activate important components of the immune system such as CD8^+^ T cells and NK cells and promote the mature differentiation of B cells and DC cells (62). NK cells and CD8^+^ T cells can inhibit tumor cell metastasis (63); hence, IFN-α can regulate the immune system to play an antitumor role. The secretion of TNF-α, IFN-γ, IL-6, and other cytokines by CD4^+^ T cells can change the TME (64, 65), induce the local invasion of T lymphocytes into the tumor (66), inhibit the synthesis of DNA and RNA of tumor cells, and thus, induce the apoptosis of tumor cells (65).

In order to further improve the overall survival (OS) rate of CRC, immunotherapy has gradually attracted our attention. For example, the gradual discovery of immune checkpoints such as PD-1, PD-L1, CTLA-4, and OX40 has led to the emergence of immune checkpoint inhibitors for CRC therapy (67). There are significant differences in treatment outcomes among different subtypes of CRC. For example, immune checkpoint inhibitors represented by the anti-PD-1/PD-L1 pathway have achieved the most outstanding curative effect in the treatment of CRC with deficient mismatch repair (dM-MR) or microsatellite instability-high (MSI-H) and have been used for the second-line treatment (68). However, most metastatic CRC patients represented by the proficiency of mismatch repair (pMMR) or microsatellite stability (MSS) not only do not respond to the above treatments; moreover, it also leads to the progression of the disease, and some patients even have adverse events related to immunotherapy such as colitis, pneumonia, dermatitis, and endocrine diseases after receiving the treatment (69, 70). At the same time, the TME is also closely related to the effect of immunotherapy. A study has shown that the accumulation of memory B cells and T cells in the TME can not only determine the clinical stage of CRC but also indicate the effect of the immune system’s antitumor response (71). TIL is characterized by tumor invasion and lymph node metastasis. As a signal of tumor cells attacking the human immune system, TIL reflects the immune response of the host (72, 73). The antitumor effect of TIL can be affected by co-inhibitory immune checkpoints and can be used as a biomarker to evaluate and predict the effect of immune checkpoint inhibitors (74, 75). CAFs that are abundant in CRC and accumulate substantially in the TME are usually associated with poor prognosis (76, 77). CAFs are positively related to PD-L1 expression in CRC tissues, and by secreting CXCL5, CAFs could promote the expression of PD-L1 in cancer cells (78).

In general, immunotherapy has opened a new chapter in cancer treatment and greatly improved the prognosis of CRC, but the therapeutic effect varies greatly among different subtypes of CRC. Therefore, it is very important to distinguish sensitive and insensitive populations by specific biomarkers. In the era of precision cancer therapy, the CRC typing system we identified has great potential to be used to predict and evaluate the effects of immunotherapy on CRC patients. In this study, we integrated the TME cells of CRC to identify four immune clusters (ImmClust-CS1, ImmClust-CS2, ImmClust-CS3, and ImmClust-CS4), which were validated using data from the GEO datasets. The four ImmClusts exhibited distinct TME compositions, CAFs, functional orientation, and immune checkpoints. The highest immune, stromal, and MeTIL scores were observed in CS2, in contrast to the lowest scores in CS4. CS1 may respond to immunotherapy, while CS2 may respond to immunotherapy after anti-CAFs.

Cancer develops through the gradual acquisition of somatic genetic changes, including point mutations, CNV, and fusion events that affect the function of key genes that regulate cell growth and survival. The occurrence of CRC is the cumulative result of a series of gene mutations. CRC not only mutates in well-known tumor-related genes (such as *APC*, *TP53*, *KRAS*, *PIK3CA*, *SMAD4*) but also mutates in other genes, including *SMAD2*, *CTNNB1*, *FAM123B*, *SOX9*, *ARID1A*, etc. (79, 80). The latest research showed that mutations of TP53, APC, KRAS, BRAF, and ATM cover most patients with CRC (81, 82). The top 15 markers with the highest mutation frequency among the four ImmClusts we constructed were PIK3CA, FAT4, FAT3, DNAH5, NEB, PCLO, HMCN1, AHNAK2, PCDH15, CACNA1E, DNAH8, ATM, VPS13B, DNAH2, and KMT2B. These mutation markers may serve as novel molecular targets for the detection or therapy of these ImmClusts. When every cell divides, it acquires random somatic mutations, and only driver mutations lead to malignant development. PIK3CA was previously defined as a driving mutation in CRC (46). Recent studies have found that mutation of PIK3CA can lead to continuous activation of the EGFR signaling pathway, thus affecting the therapeutic efficiency of anti-EGFR drugs (83). Ejima et al. found high-frequency mutations in ATM introns in CRC cell lines (84). Genes with different mutation frequencies are expected to be markers for the detection or treatment of subtypes. Among the four ImmClusts, the top 15 markers with the highest mutation frequency were acquired, and CS1 had significantly lower CNA at the focal level than other subtypes. In addition, CS1 and CS2 patients had more stable chromosomes than CS3 and CS4. The most sensitive chemotherapeutic agents in these four ImmClusts were also found.

To sum up, the high immune infiltration, low fibroblast infiltration, high mutation load, and low chromosomal variation of CS1 are related to the ability of this subtype to respond to immunotherapy.

Our study was the first one to establish the cluster system based on TME for CRC. The combined analysis of data from the TCGA and GEO verified the accuracy of the classification system. Nevertheless, the clustering system constructed by us lacks large prospective studies to verify, and its specificity and sensitivity need to be further determined.

## Conclusions

This work revealed the novel clusters based on the TME for CRC, which would guide in predicting the prognosis, biological features, and appropriate treatment for patients with CRC.

## Data availability statement

The datasets presented in this study can be found in online repositories. The names of the repository/repositories and accession number(s) can be found in the article/**Supplementary Material**.

## Ethics statement

This study was reviewed and approved by The study was approved by the ethics committee of the First Affiliated Hospital of Zhengzhou University (Ethics No. 2021-KY-0147-002). The patients/participants provided their written informed consent to participate in this study.

## Author contributions

All authors contributed to the article and approved the submitted version.

## Funding

This study was supported by the Collaborative Innovation Major Project of Zhengzhou (Grant No. 20XTZX08017), the Scientific and Technological Project of Henan Province (No. 2018020578), the National Natural Science Foundation of China (Grant No. 82002433), the Science and Technology Project of Henan Provincial Department of Education (Grant No. 21A320036), and the Young and Middle-aged Health Science and Technology Innovation Talents in 2020 (Grant No. YXKC2020049).

## Conflict of interest

The authors declare that the research was conducted in the absence of any commercial or financial relationships that could be construed as a potential conflict of interest.

## Publisher’s note

All claims expressed in this article are solely those of the authors and do not necessarily represent those of their affiliated organizations, or those of the publisher, the editors and the reviewers. Any product that may be evaluated in this article, or claim that may be made by its manufacturer, is not guaranteed or endorsed by the publisher.

## Abbreviations

CRC, colorectal cancer; TME, tumor microenvironment; TCGA, The Cancer Genome Atlas; GEO, Gene Expression Omnibus; MeTIL, methylation of tumor-infiltrating lymphocyte; ImmClust, immune clusters; CAF, cancer-associated fibroblast; TGF-β, transforming growth factor-β; TAM, tumor-associated macrophage; TIL, tumor-infiltrating lymphocyte; CMS, consensus molecular subtype; MSI, microsatellite instability; FPKM, fragments per kilobase million; CNV, copy number variation; PCA, principal component analysis; ICB, immune checkpoint blockade; SCNA, somatic copy number variation; GDSC, Genomics of Drug Sensitivity in Cancer; EMT, epithelial–mesenchymal transition; IES, immune enrichment score; CRG, cancer-associated fibroblast-related gene; OS, overall survival; dM-MR, deficient mismatch repair; MSI-H, microsatellite instability-high; pMMR, proficiency of mismatch repair; MSS, microsatellite stability.
