# Supplementary material for: Identification and validation of immunotherapy for four novel clusters of colorectal cancer based on the tumor microenvironment
Source: Front Immunol. 2022 Oct 28;13:984480. doi: 10.3389/fimmu.2022.984480 (PMC9650243; doi:10.3389/fimmu.2022.984480)
Supplement: Supplementary file 1 [file DataSheet_1.docx]

display.progress = function (index, totalN, breakN=20) {

if ( index %% ceiling(totalN/breakN) ==0 ) {

cat(paste(round(index*100/totalN), "% ", sep=""))

}

}

standarize.fun <- function(indata=NULL, halfwidth=NULL, centerFlag=T, scaleFlag=T) {

outdata=t(scale(t(indata), center=centerFlag, scale=scaleFlag))

if (!is.null(halfwidth)) {

outdata[outdata>halfwidth]=halfwidth

outdata[outdata<(-halfwidth)]= -halfwidth

}

return(outdata)

}

gmt2list <- function(annofile){

if (!file.exists(annofile)) {

stop("There is no such gmt file.")

}

if (tools::file_ext(annofile) == "xz") {

annofile <- xzfile(annofile)

x <- scan(annofile, what="", sep="\n", quiet=TRUE)

close(annofile)

} else if (tools::file_ext(annofile) == "gmt") {

x <- scan(annofile, what="", sep="\n", quiet=TRUE)

} else {

stop ("Only gmt and gmt.xz are accepted for gmt2list")

}

y <- strsplit(x, "\t")

names(y) <- sapply(y, `[[`, 1)

annoList <- lapply(y, `[`, c(-1,-2))

}

countToFpkm <- function(counts, effLen){

N <- sum(counts)

exp( log(counts) + log(1e9) - log(effLen) - log(N) )

}

fpkmToTpm <- function(fpkm)

{

exp(log(fpkm) - log(sum(fpkm)) + log(1e6))

}

# set colors

blue <- "#5bc0eb"

yellow <- "#fde74c"

green <- "#9bc53d"

red <- "#f25f5c"

purple <- "#531f7a"

grey <- "#8693ab"

orange <- "#fa7921"

white <- "#f2d7ee"

darkred <- "#F2042C"

lightred <- "#FF7FBF"

lightblue <- "#B2EBFF"

darkblue <- "#1d00ff"

cherry <- "#700353"

lightgrey <- "#dcddde"

nake <- "#F8C364"

gold <- "#ECE700"

cyan <- "#00B3D0"

sun <- "#E53435"

peach <- "#E43889"

violet <- "#89439B"

soil <- "#EC7D21"

lightgreen <- "#54B642"

darkblue <- "#21498D"

darkgreen <- "#009047"

brown <- "#874118"

seagreen <- "#008B8A"

jco <- c("#2874C5","#EABF00","#868686","#C6524A","#80A7DE")

jama <- c("#3B4E55","#D69044","#44A0D5","#A94747","#81AF96","#6A6599","#80796B")

npg <- c("#E64B35","#4DBBD5","#00A087","#3C5488")

heatmap.BlWtRd <- c("#6699CC","white","#FF3C38")

heatmap.YlGnPe <- c("#440259","#345F8C","#228C8A","#78CE51","#FAE71F")

heatmap.GrWtRd <- c("#2b2d42","#8d99ae","#edf2f4","#ef233c","#d90429")

heatmap.L.BlYlRd <- c("#4281a4","#9cafb7","#ead2ac","#e6b89c","#fe938c")

heatmap.BlBkRd <- c("#54FEFF","#32ABAA","#125456","#000000","#510000","#A20000","#F30000")

heatmap.BlWtRd2 <- c("#183869","#4195C1","white","#CB5746","#62011D")

heatmap.fancy <- c("#10040A", "#2A0B35", "#4D155B", "#73215B", "#9C3558", "#C34D44", "#E07038", "#F2981C", "#F2CA51", "#FAF6A3")

clust.col <- c("#DD492E","#40548A","#32A087","#EC7D21")

heatmap.meth <- c("#183869","#4195C1","green","yellow","#CB5746","#62011D")

mycol <- brewer.pal(12, "Paired")

#-----------------#

# data processing #

# load MSI information

load(file.path(data.path,"msi_results.rda"))

rownames(msi_results) <- paste0(msi_results$sample,"-01A")

# load phenotype information, downloaded from https://xenabrowser.net/datapages/

sinfo.coad <- read.delim(file.path(data.path,"TCGA-COAD.GDC_phenotype.tsv"), sep = "\t", row.names = 1, check.names = F, stringsAsFactors = F, header = T)

sinfo.read <- read.delim(file.path(data.path,"TCGA-READ.GDC_phenotype.tsv"), sep = "\t", row.names = 1, check.names = F, stringsAsFactors = F, header = T)

comindicator <- intersect(colnames(sinfo.coad), colnames(sinfo.read))

sinfo <- rbind.data.frame(sinfo.coad[,comindicator],

sinfo.read[,comindicator])

# extract variable of interest

clincvar <- c("disease_code",

"submitter_id",

"age_at_initial_pathologic_diagnosis",

"gender.demographic",

"colon_polyps_present",

"lymphatic_invasion",

"pathologic_T",

"pathologic_N",

"pathologic_M",

"tumor_stage.diagnoses",

"perineural_invasion_present",

"disease_type",

"primary_site",

"tumor_grade.diagnoses")

sinfo <- sinfo[,clincvar]

# extract Adenomas and Adenocarcinomas

sinfo <- sinfo[which(sinfo$disease_type == "Adenomas and Adenocarcinomas"),]

# extract primary tumors of COADREAD

sinfo <- sinfo[substr(rownames(sinfo),14,16) == "01A",]

# COAD READ

# 390 154

# load survival data from pancancer atlas, downloaded from https://gdc.cancer.gov/about-data/publications/pancanatlas

surv <- read.delim(file.path(data.path,"pancancerSurvivalData_XLu.txt"),sep = "\t",row.names = 1,check.names = F,stringsAsFactors = F,header = T)

surv <- surv[,c("OS","OS.time","PFI","PFI.time")]

surv$submitter_id <- rownames(surv)

# add survival data to sinfo

sinfo$barcode <- rownames(sinfo)

sinfo <- merge(sinfo, surv, by = "submitter_id", all.x = TRUE)

rownames(sinfo) <- sinfo$barcode

sinfo <- sinfo[!is.na(sinfo$OS),] # 540, 388+152

# load expression data and combined CPAD with READ

fpkm.coad <- read.delim(file.path(data.path,"TCGA-COAD.htseq_fpkm.tsv.gz"), sep = "\t", row.names = 1, check.names = F, stringsAsFactors = F, header = T)

fpkm.coad <- 2^fpkm.coad - 1

fpkm.read <- read.delim(file.path(data.path,"TCGA-READ.htseq_fpkm.tsv.gz"), sep = "\t", row.names = 1, check.names = F, stringsAsFactors = F, header = T)

fpkm.read <- 2^fpkm.read - 1

fpkm <- cbind.data.frame(fpkm.coad,fpkm.read)

# convert fpkms to tpm

tpm <- apply(fpkm, 2, fpkmToTpm)

tpm <- as.data.frame(log2(tpm + 1))

rm(fpkm); gc()

# load gene information

Ginfo <- read.delim(file.path(comAnn.path,"gencode.v22.annotation.gene.probeMap"),row.names = 1,sep = "\t",check.names = F,stringsAsFactors = F,header = T)

comgene <- intersect(rownames(Ginfo), rownames(tpm))

Ginfo <- Ginfo[comgene,]

tpm <- tpm[comgene,]

identical(rownames(tpm), rownames(Ginfo))

tpm$Gene <- Ginfo[rownames(tpm),"gene"]

tpm <- as.data.frame(apply(tpm[,setdiff(colnames(tpm), "Gene")], 2, function(x) tapply(x, INDEX = factor(tpm$Gene), FUN=median, na.rm = TRUE))) # take median value for multiple match

overlapTab <- read.table(file.path(comAnn.path,"overlapTable_hg38.txt"),sep = "\t",row.names = 1,check.names = F,stringsAsFactors = F,header = T)

Mids <- intersect(overlapTab[which(overlapTab$genetype == "protein_coding"),"genename"], rownames(tpm))

Lids <- intersect(overlapTab[which(overlapTab$genetype == "lncRNA"),"genename"], rownames(tpm))

# extract tumor and normal samples from expression data

tum.sam.expr <- colnames(tpm)[substr(colnames(tpm),14,16) == "01A"] # 616 coadread

tum.sam.expr <- intersect(rownames(sinfo), tum.sam.expr) # 530 coadread

nor.sam.expr <- colnames(tpm)[substr(colnames(tpm),14,16) == "11A"] # 51

# remove batch effect

batch <- data.frame(batch = rep(c("COAD","READ"),

times = c(ncol(fpkm.coad),

ncol(fpkm.read))))

modcombat <- model.matrix(~1, data = batch)

tpm.combat <- as.data.frame(ComBat(dat=as.matrix(tpm), batch=batch$batch, mod=modcombat))

write.table(tpm.combat, file = file.path(data.path,"TCGA_TPM_COMBAT.txt"),sep = "\t",row.names = T,col.names = NA,quote = F)

batchPCA(indata = t(scale(t(tpm))),

batch = batch$batch,

fig.dir = fig.path,

PCA.fig.title = "PCA for COADREAD combined expression profile before ComBat",

cols = npg[1:2],

showID = F,

cex = 0.9,

showLegend = T)

batchPCA(indata = t(scale(t(tpm.combat))),

batch = batch$batch,

fig.dir = fig.path,

PCA.fig.title = "PCA for COADREAD combined expression profile after ComBat",

cols = npg[1:2],

showID = F,

cex = 0.9,

showLegend = T)

# create annotation information

annCol <- data.frame(row.names = tum.sam.expr,

Disease = sinfo[tum.sam.expr,"disease_code"],

Age = sinfo[tum.sam.expr,"age_at_initial_pathologic_diagnosis"],

Sex = sinfo[tum.sam.expr,"gender.demographic"],

pStage = sinfo[tum.sam.expr,"tumor_stage.diagnoses"],

tStage = sinfo[tum.sam.expr,"pathologic_T"],

nStage = sinfo[tum.sam.expr,"pathologic_N"],

mStage = sinfo[tum.sam.expr,"pathologic_M"],

lymphatic_invasion = sinfo[tum.sam.expr,"lymphatic_invasion"],

colon_polyps = sinfo[tum.sam.expr,"colon_polyps_present"],

perineural_invasion = sinfo[tum.sam.expr,"perineural_invasion_present"],

primary_site = sinfo[tum.sam.expr,"primary_site"],

OS = sinfo[tum.sam.expr,"OS"],

PFS = sinfo[tum.sam.expr,"PFI"],

stringsAsFactors = F)

annCol$Age <- ifelse(annCol$Age > median(annCol$Age), ">66.5","<=66.5")

annCol[annCol == ""] <- "Missing"

table(annCol$pStage)

annCol[which(annCol$pStage == "not reported"), "pStage"] <- "Missing"

annCol[which(annCol$pStage %in% c("stage i","stage ia")), "pStage"] <- "stage i"

annCol[which(annCol$pStage %in% c("stage ii","stage iia","stage iib","stage iic")), "pStage"] <- "stage ii"

annCol[which(annCol$pStage %in% c("stage iii","stage iiia","stage iiib","stage iiic")), "pStage"] <- "stage iii"

annCol[which(annCol$pStage %in% c("stage iv","stage iva","stage ivb")), "pStage"] <- "stage iv"

table(annCol$tStage)

annCol[which(annCol$tStage %in% c("T4","T4a","T4b")), "tStage"] <- "T4"

annCol[which(annCol$tStage %in% c("Tis")), "tStage"] <- "Missing"

table(annCol$nStage)

annCol[which(annCol$nStage %in% c("N1","N1a","N1b","N1c")), "nStage"] <- "N1"

annCol[which(annCol$nStage %in% c("N2","N2a","N2b")), "nStage"] <- "N1"

annCol[which(annCol$nStage %in% c("NX")), "nStage"] <- "Missing"

table(annCol$mStage)

annCol[which(annCol$mStage %in% c("MX")), "mStage"] <- "Missing"

annCol[which(annCol$mStage %in% c("M1","M1a","M1b")), "mStage"] <- "M1"

table(annCol$lymphatic_invasion)

table(annCol$colon_polyps)

table(annCol$perineural_invasion)

table(annCol$primary_site)

table(annCol$OS)

table(annCol$PFS)

annCol$OS <- ifelse(annCol$OS == "1", "Dead", "Alive")

annCol$PFS <- ifelse(annCol$PFS == "1", "Progression", "Progression-free")

annCol$pStage <- factor(annCol$pStage, levels = c("stage i","stage ii","stage iii","stage iv","Missing"))

annCol$tStage <- factor(annCol$tStage, levels = c("T1","T2","T3","T4","Missing"))

annCol$nStage <- factor(annCol$nStage, levels = c("N0","N1","Missing"))

annCol$mStage <- factor(annCol$mStage, levels = c("M0","M1","Missing"))

annCol$lymphatic_invasion <- factor(annCol$lymphatic_invasion, levels = c("YES","NO","Missing"))

annCol$colon_polyps <- factor(annCol$colon_polyps, levels = c("YES","NO","Missing"))

annCol$perineural_invasion <- factor(annCol$perineural_invasion, levels = c("YES","NO","Missing"))

annCol$primary_site <- factor(annCol$primary_site, levels = c("Colon","Rectum","Rectosigmoid junction","Connective, subcutaneous and other soft tissues"))

annCol$OS <- factor(annCol$OS, levels = c("Alive","Dead"))

annCol$PFS <- factor(annCol$PFS, levels = c("Progression-free","Progression"))

annColors <- list()

annColors[["Disease"]] <- c("COAD" = npg[1],"READ" = npg[2])

annColors[["Age"]] <- c("<=66.5" =seagreen,">66.5" = orange)

annColors[["Sex"]] <- c("female" = lightred, "male" = "skyblue")

annColors[["pStage"]] <- c("stage i" = mycol[2], "stage ii" = mycol[1], "stage iii" = mycol[5], "stage iv" = mycol[6], "Missing" = "white")

annColors[["tStage"]] <- c("T1" = mycol[4], "T2" = mycol[3], "T3" = mycol[7], "T4" = mycol[8], "Missing" = "white")

annColors[["nStage"]] <- c("N0" = mycol[9], "N1" = mycol[10], "Missing" = "white")

annColors[["mStage"]] <- c("M0" = mycol[11], "M1" = mycol[12], "Missing" = "white")

annColors[["lymphatic_invasion"]] <- c("YES" = purple, "NO" = "grey90", "Missing" = "white")

annColors[["colon_polyps"]] <- c("YES" = purple, "NO" = "grey90", "Missing" = "white")

annColors[["perineural_invasion"]] <- c("YES" = purple, "NO" = "grey90", "Missing" = "white")

annColors[["OS"]] <- c("Dead" = "black", "Alive" = "grey90")

annColors[["PFS"]] <- c("Progression" = "black", "Progression-free" = "grey90")

annColors[["primary_site"]] <- c("Colon" = npg[1], "Rectum" = npg[2],"Rectosigmoid junction" = npg[3],"Connective, subcutaneous and other soft tissues" = npg[4])

#--------------#

# estimate TME #

mcp.coadread <- MCPcounter.estimate(as.matrix(tpm.combat[,tum.sam.expr]),

featuresType = "HUGO_symbols",

genes = read.table(file.path(comAnn.path,"genes.txt"),sep = "\t",stringsAsFactors = F,header = T,colClasses = "character",check.names = F),

probesets = read.table(file.path(comAnn.path,"probesets.txt"),sep = "\t",stringsAsFactors = F,header = F,colClasses = "character",check.names = F))

indata <- tpm.combat[,tum.sam.expr]

write.table(indata,file = file.path(res.path,"TCGA_log2TPM_hugo.txt"),sep = "\t",row.names = T,col.names = NA,quote = F)

filterCommonGenes(input.f=file.path(res.path, "TCGA_log2TPM_hugo.txt") , output.f=file.path(res.path,"TCGA_log2TPM_hugo_ESTIMATE.txt"), id="GeneSymbol")

estimateScore(file.path(res.path,"TCGA_log2TPM_hugo_ESTIMATE.txt"), file.path(res.path,"TCGA_log2TPM_hugo_estimate_score.txt"), platform="affymetrix")

est.tcga <- read.table(file = file.path(res.path,"TCGA_log2TPM_hugo_estimate_score.txt"),header = T,row.names = NULL,check.names = F,stringsAsFactors = F,sep = "\t")

rownames(est.tcga) <- est.tcga[,2]; colnames(est.tcga) <- est.tcga[1,]; est.tcga <- est.tcga[-1,c(-1,-2)];

est.tcga <- sapply(est.tcga, as.numeric); rownames(est.tcga) <- c("StromalScore","ImmuneScore","ESTIMATEScore","TumorPurity"); est.tcga.backup = as.data.frame(est.tcga); colnames(est.tcga.backup) <- colnames(indata)

est.tcga <- annTrackScale(indata = est.tcga, halfwidth = 2, poolsd = F); est.tcga <- as.data.frame(t(est.tcga))

rownames(est.tcga) <- tum.sam.expr

#annCol$TP <- as.numeric(est.tcga.backup[4,rownames(annCol)])

annCol$IES <- est.tcga[rownames(annCol),"ImmuneScore"]

annCol$SES <- est.tcga[rownames(annCol),"StromalScore"]

annColors[["IES"]] <- annColors[["SES"]] <- viridis::inferno(64)

#annColors[["TP"]] <- NMF:::ccRamp(c("grey80","black"),64)

# purify

# tum.sam.expr.tp <- rownames(annCol[which(annCol$TP < 0.95),])

# mcp.coadread.pure <- mcp.coadread[,tum.sam.expr.tp]

# for (sam in colnames(mcp.coadread.pure)) {

# for (cell in rownames(mcp.coadread.pure)) {

# mcp.coadread.pure[cell,sam] <- mcp.coadread[cell,sam]/(1-est.tcga.backup[4,sam])

# }

# }

# other signature

immunosuppression.signature <- c("CXCL12","TGFB1","TGFB3","LGALS1")

t.cell.activation.signature <- c("CXCL9","CXCL10","CXCL16","IFNG","IL15")

t.cell.survival.signature <- c("CD70","CD27")

regulatory.t.cell.signature <- c("FOXP3","TNFRSF18")

mhc.signature <- c("HLA-A","HLA-B","HLA-E","HLA-F","HLA-G","B2M")

myeloid.signature <- "CCL2"

tls.signature <- "CXCL13"

# immune checkpoint gene

ici.gene <- c("PDCD1","CD274","PDCD1LG2","CTLA4","HAVCR2","LAG3")

# check

is.element(immunosuppression.signature, rownames(tpm.combat))

is.element(t.cell.activation.signature, rownames(tpm.combat))

is.element(t.cell.survival.signature, rownames(tpm.combat))

is.element(regulatory.t.cell.signature, rownames(tpm.combat))

is.element(mhc.signature, rownames(tpm.combat))

is.element(myeloid.signature, rownames(tpm.combat))

is.element(tls.signature, rownames(tpm.combat))

is.element(ici.gene, rownames(tpm.combat))

geoMean <- function(x) {exp(mean(log(x)))}

tmp <- as.data.frame(pmax(as.matrix(tpm.combat),0) * 2)

immunosuppression.geoMean <- apply(tmp[immunosuppression.signature,], 2, geoMean)

t.cell.activation.geoMean <- apply(tmp[t.cell.activation.signature,], 2, geoMean)

t.cell.survival.geoMean <- apply(tmp[t.cell.survival.signature,], 2, geoMean)

regulatory.t.cell.geoMean <- apply(tmp[regulatory.t.cell.signature,], 2, geoMean)

mhc.geoMean <- apply(tmp[mhc.signature,], 2, geoMean)

myeloid.geoMean <- apply(tmp[myeloid.signature,], 2, geoMean)

tls.geoMean <- apply(tmp[tls.signature,], 2, geoMean)

## mutation

maf <- read_tsv(file.path(data.path,"data_mutations.txt"), comment = "#")

label <- c("Tumor_Sample_Barcode",

"Hugo_Symbol",

"Chromosome",

"Start_Position",

"End_Position",

"Variant_Classification",

"Variant_Type",

"Reference_Allele",

"Tumor_Seq_Allele1",

"Tumor_Seq_Allele2")

maf <- maf[,label]

maf$Hugo_Symbol <- toupper(maf$Hugo_Symbol) # transfer gene name to capital. (eg, C1orf198 to C1ORF198)

write.table(maf, file.path(data.path,"TCGA_COADREAD_MAF.txt"), quote=F, row.names=F,col.names = T,sep = "\t")

mut.binary <- matrix(0,nrow = length(unique(maf$Hugo_Symbol)),ncol = length(unique(maf$Tumor_Sample_Barcode)),dimnames = list(unique(maf$Hugo_Symbol),unique(maf$Tumor_Sample_Barcode)))

for (i in colnames(mut.binary)) {

tmp <- maf[which(maf$Tumor_Sample_Barcode == i),]

tmp <- tmp[which(tmp$Variant_Classification %in% c("Frame_Shift_Del", "Frame_Shift_Ins", "Splice_Site", "Translation_Start_Site","Nonsense_Mutation", "Nonstop_Mutation", "In_Frame_Del","In_Frame_Ins", "Missense_Mutation")),]

for (j in tmp$Hugo_Symbol)

mut.binary[j,i] <- 1

}

mut.binary <- as.data.frame(mut.binary)#; rownames(mut.binary) <- toupper(rownames(mut.binary))

mut.binary <- mut.binary[rowSums(mut.binary) > 0,]

mut.binary <- mut.binary[,which(substr(colnames(mut.binary), 14, 15) == "01")]

colnames(mut.binary) <- paste0(colnames(mut.binary),"A")

tum.sam.common <- intersect(tum.sam.expr, colnames(mut.binary))

# classification using mcpcounter

indata <- t(scale(t(mcp.coadread[,tum.sam.expr])))

hcs <- hclust(distanceMatrix(as.matrix(indata), "euclidean"), "ward.D2") # 原文提到使用欧式距离以及ward's聚类方式

group <- cutree(hcs, k = 5)

group <- paste0("CS", group); names(group) <- colnames(mcp.coadread)

group <- sapply(group,function(x) {

switch(x,

"CS1" = "CS3",

"CS2" = "CS4",

"CS3" = "CS4",

"CS4" = "CS2",

"CS5" = "CS1")})

annCol$ImmClust <- group[rownames(annCol)]

annColors[["ImmClust"]] <- c("CS1" = mycol[7],"CS2" = mycol[5],"CS3" = mycol[3],"CS4" = mycol[1])

annCol <- annCol[order(annCol$ImmClust),]

tme.dat <- data.frame("Immunosuppression" = immunosuppression.geoMean,

"T cell activation" = t.cell.activation.geoMean,

"T cell survival" = t.cell.survival.geoMean,

"Regulatory T cell" = regulatory.t.cell.geoMean,

"Class I MHC" = mhc.geoMean,

"Myeloid cells chemotactism" = myeloid.geoMean,

"TLSs" = tls.geoMean)

# tide

tcga.tide <- tpm.combat

tcga.tide <- tcga.tide[rowSums(tcga.tide) > 0,]

tcga.tide <- sweep(tcga.tide[,rownames(annCol)],1,apply(tcga.tide[,nor.sam.expr], 1, median))

#tcga.tide <- sweep(tcga.tide,2, apply(tcga.tide,2,median,na.rm=T))

#tcga.tide <- round(sweep(tcga.tide,1, apply(tcga.tide,1,median,na.rm=T)),3)

write.table(tcga.tide,file.path(res.path,"tcga.tide.input.txt"),sep = "\t",row.names = T,col.names = NA,quote = F)

tide.res <- read.csv(file.path(res.path,"tcga.tide.output.csv"),row.names = 1,check.names = F,stringsAsFactors = F,header = T)

tide.res$ImmClust <- annCol[rownames(tide.res),"ImmClust"]

table(tide.res$ImmClust, tide.res$Responder)

boxplot(tide.res$TIDE~tide.res$ImmClust)

pairwise.wilcox.test(tide.res$TIDE,tide.res$ImmClust)

annCol$Responder <- tide.res[rownames(annCol),"Responder"]

annCol$TIDE <- tide.res[rownames(annCol),"TIDE"]

annColors[["Responder"]] <- c("True" = purple,"False" = "grey90")

annColors[["TIDE"]] <- bluered(64)

## MCPcounter heatmap

indata <- mcp.coadread

plotdata <- standarize.fun(indata, halfwidth = 2)

hm1 <- pheatmap(plotdata[,rownames(annCol)],

border_color = NA, # 无边框

color = bluered(64), # 采用红蓝颜色

show_rownames = T, # 显示行名

show_colnames = F, # 不显示列名

cluster_rows = F, # 行不聚类

cluster_cols = F, # 列不聚类

name = "Gene/metagene\nZ-score", # 颜色图例的名字（所有热图统一，这样只会显示一个）

cellheight = 12, # 热图元素高度

cellwidth = 300/ncol(plotdata), # 热图总宽度固定为300

annotation_col = annCol[,c("ImmClust","Disease","Age","Sex","tStage","nStage","mStage","lymphatic_invasion","colon_polyps","perineural_invasion","primary_site","IES","SES","TIDE","Responder")], # 样本注释

annotation_colors = annColors[c("ImmClust","Disease","Age","Sex","tStage","nStage","mStage","lymphatic_invasion","colon_polyps","perineural_invasion","primary_site","IES","SES","TIDE","Responder")]) # 注释颜色

## other signature heatmap

plotdata <- standarize.fun(t(tme.dat[rownames(annCol),]), halfwidth = 2)

hm2 <- pheatmap(plotdata,

border_color = NA,

color = bluered(64),

show_rownames = T,

show_colnames = F,

cluster_rows = F,

cluster_cols = F,

name = "Gene/metagene\nZ-score",

cellheight = 12,

cellwidth = 300/ncol(plotdata))

## ici heatmap

plotdata <- standarize.fun(tpm.combat[ici.gene,rownames(annCol)], halfwidth = 2)

hm3 <- pheatmap(plotdata,

border_color = NA,

color = bluered(64),

show_rownames = T,

show_colnames = F,

cluster_rows = F,

cluster_cols = F,

name = "Gene/metagene\nZ-score",

cellheight = 12,

cellwidth = 300/ncol(plotdata))

pdf(file = file.path(fig.path,"tme heatmap of tcga.pdf"), width = 10,height = 12)

draw(hm1 %v% hm2 %v% hm3,

heatmap_legend_side = "bottom",

annotation_legend_side = "bottom")

invisible(dev.off())

# submap

generateInputFileForSubMap <- function(in_gct, gct_file, cls_file, sam_info, type_name = "type"){

in_gct <- data.frame(GeneID=rownames(in_gct),

description="na",

in_gct,

stringsAsFactors = F,

check.names = F)

cat("#1.2\n", file = gct_file)

cat(nrow(in_gct),"\t",ncol(in_gct)-2,"\n", file = gct_file, append = T)

cat(paste(colnames(in_gct), collapse = "\t"),"\n", file = gct_file, append = T)

for(i in 1:nrow(in_gct)) cat(paste(in_gct[i,], collapse = "\t"),"\n", file = gct_file, append = T)

cat(nrow(sam_info),length(levels(factor(sam_info$rank))),1, "\n", file = cls_file )

cat("#", paste0(levels(factor(sam_info[, type_name])), collapse = " " ), "\n", file = cls_file, sep = "", append = T)

cat(as.numeric(factor(sam_info[, type_name])), file = cls_file, append = T)

}

skcm.immunotherapy.logNC <- read.table(file.path(data.path,"skcm.immunotherapy.47samples.log2CountsNorm.txt"),sep = "\t",row.names = 1,header = T,check.names = F,stringsAsFactors = F) #鍘熸枃鎻愪緵鐨刲og2杞寲鐨勬爣鍑嗗寲count鍊?

rownames(skcm.immunotherapy.logNC) <- toupper(rownames(skcm.immunotherapy.logNC)) # 鍩哄洜澶у啓锛屽洜涓烘垜浣跨敤鐨勬暟鎹槸鎶婂熀鍥犲悕閮藉ぇ鍐欑殑

skcm.immunotherapy.info <- read.table(file.path(data.path,"skcm.immunotherapy.47sampleInfo.txt"),sep = "\t",row.names = 1,header = T,check.names = F,stringsAsFactors = F)

skcm.immunotherapy.info <- skcm.immunotherapy.info[order(skcm.immunotherapy.info$label),]

skcm.immunotherapy.info$rank <- rep(c(1,2,3,4),times=as.character(table(skcm.immunotherapy.info$label))) #1: CTLA4_noR 2: CTLA4_R 3:PD1_noR 4:PD1_R

sam_info <- skcm.immunotherapy.info

GENELIST <- intersect(rownames(tpm.combat),rownames(skcm.immunotherapy.logNC))

in_gct <- skcm.immunotherapy.logNC[GENELIST,rownames(skcm.immunotherapy.info)]

gct_file <- file.path(res.path,"skcm.immunotherapy.for.SubMap.gct")

cls_file <- file.path(res.path,"skcm.immunotherapy.for.SubMap.cls")

generateInputFileForSubMap(in_gct = in_gct, gct_file = gct_file, cls_file = cls_file, sam_info = sam_info, type_name = "rank")

# tcga

sam_info <- data.frame("ImmClust"=annCol$ImmClust,row.names = rownames(annCol))

sam_info$rank <- as.numeric(gsub("CS","",sam_info$ImmClust))

sam_info <- sam_info[order(sam_info$rank),]

gct_file <- file.path(res.path,"tcga.immclust.for.SubMap.gct")

cls_file <- file.path(res.path,"tcga.immclust.for.SubMap.cls")

in_gct <- tpm.combat[GENELIST,rownames(sam_info)]

generateInputFileForSubMap(in_gct = in_gct, gct_file = gct_file, cls_file = cls_file, sam_info = sam_info, type_name = "rank")

# submap heatmap

tmp <- matrix(c(0.868,0.265,0.934,0.001, # nominal p value

0.566,0.073,0.848,0.001,

0.063,0.028,0.247,0.815,

0.551,0.852,0.551,0.885,

1,1,1,0.016, # Bonferroni adjusted p value

1,1,1,0.016,

1,0.448,1,1,

1,1,1,1,

0.992,0.605,0.934,0.011, # FDR adjusted p value

0.906,0.233,1,0.011,

0.252,0.149,0.658,1,

1,1,1,0.944),

nrow = 12,byrow = T,

dimnames = list(c("CS1","CS2","CS3","CS4"," CS1"," CS2"," CS3"," CS4"," CS1"," CS2"," CS3"," CS4"),c("CTAL4-noR","CTLA4-R","PD1-noR","PD1-R")))

hm <- pheatmap(t(tmp),

border_color = "white",

number_format = "%.3f",

cellwidth = 30, cellheight = 30,

cluster_rows = F,cluster_cols = F,

color = rev(NMF:::ccRamp(c("#E6EAF7","#B6D1E8","#498EB9","#204F8D"),64)),

display_numbers = T,

number_color = "black",

fontsize_number = 9,

name = "Statitic",

annotation_col = data.frame(pvalue=c("Nominal p value","Nominal p value","Nominal p value","Nominal p value","Bonferroni adjusted","Bonferroni adjusted","Bonferroni adjusted","Bonferroni adjusted","FDR adjusted","FDR adjusted","FDR adjusted","FDR adjusted"),

row.names = rownames(tmp)),

annotation_colors = list(pvalue=c("Nominal p value"="black","Bonferroni adjusted"="grey50","FDR adjusted" = "grey80")))

pdf(file.path(fig.path,"submap heatmap of predicted response to immunotherapy in tcga coadread.pdf"),width = 10,height = 4)

draw(hm, heatmap_legend_side = "left",annotation_legend_side = "bottom")

invisible(dev.off())

# caf signature

caf.gene <- c("ACTA2","PDGFRA","PDGFRB","THY1","COL1A1","FAP","PDPN")

is.element(caf.gene,rownames(tpm.combat))

fibroblast.signature <- read.table(file.path(comAnn.path,"CD_Fibroblast_Signature.txt"),sep = "\t",check.names = F,stringsAsFactors = F,header = T,row.names = NULL)

cell.type <- unique(fibroblast.signature$CellType)

fibroblast.sig.cd <- list()

for (i in cell.type) {

fibroblast.sig.cd[[i]] <- fibroblast.signature[which(fibroblast.signature$CellType == i),"Symbol"]

}

fibgsva.tcga <- gsva(as.matrix(tpm.combat),fibroblast.sig.cd,method = "gsva")

indata <- standarize.fun(tpm.combat[caf.gene, rownames(annCol)], halfwidth = 2)

hm1 <- pheatmap(indata[,rownames(annCol)],

border_color = NA, # 无边框

color = greenred(64), # 采用红蓝颜色

show_rownames = T, # 显示行名

show_colnames = F, # 不显示列名

cluster_rows = F, # 行不聚类

cluster_cols = F, # 列不聚类

name = "Gene\nZ-score", # 颜色图例的名字（所有热图统一，这样只会显示一个）

cellheight = 12, # 热图元素高度

cellwidth = 300/ncol(plotdata), # 热图总宽度固定为300

annotation_col = annCol[,c("ImmClust","IES","SES","TIDE","Responder")], # 样本注释

annotation_colors = annColors[c("ImmClust","IES","SES","TIDE","Responder")]) # 注释颜色

indata <- standarize.fun(fibgsva.tcga, halfwidth = 1)

hm2 <- pheatmap(indata[,rownames(annCol)],

border_color = NA, # 无边框

color = greenred(64), # 采用红蓝颜色

show_rownames = T, # 显示行名

show_colnames = F, # 不显示列名

cluster_rows = F, # 行不聚类

cluster_cols = F, # 列不聚类

name = "GSVA", # 颜色图例的名字（所有热图统一，这样只会显示一个）

cellheight = 12, # 热图元素高度

cellwidth = 300/ncol(plotdata)) # 注释颜色

pdf(file.path(fig.path,"heatmap of caf in tcga.pdf"), width = 8, height = 8)

draw(hm1 %v% hm2, heatmap_legend_side = "bottom",annotation_legend_side = "bottom")

invisible(dev.off())

tmp <- cbind.data.frame(sinfo[rownames(annCol),],

cluster = annCol$ImmClust)

tmp$OS.time <- tmp$OS.time/30

fitd <- survdiff(Surv(OS.time, OS) ~ cluster, data=tmp, na.action=na.exclude)

p.val <- 1-pchisq(fitd$chisq, length(fitd$n)-1)

fit <- survfit(Surv(OS.time, OS)~ cluster, data=tmp, type="kaplan-meier", error="greenwood", conf.type="plain", na.action=na.exclude)

pairwise_survdiff(Surv(OS.time, OS)~ cluster,

data = tmp,

p.adjust.method = "none")

names(fit$strata) <- gsub("cluster=", "", names(fit$strata))

p <- ggsurvplot(fit, conf.int=F,risk.table=F, risk.table.col="strata",palette = mycol[c(7,5,3,1)],

pval = F,data=tmp,size=1,

tables.height = 0.3,surv.median.line = "hv",

xlab = "Time (Months)",ylab = "OS",

ylim = c(0,1),

risk.table.y.text = F)

p.lab <- paste0("Overall P",

ifelse(p.val < 0.001, " < 0.001",

paste0(" = ",round(p.val, 3))))

p$plot <- p$plot + annotate("text",

x = 0, y = 0.25,

hjust = 0,

fontface = 4,

label = p.lab)

pdf.options(reset = TRUE, onefile = FALSE)

pdf(file.path(fig.path,"km of os using immune cluster in coadread of tcga.pdf"),width = 5,height = 5)

print(p)

dev.off()

# load external cohort

## gse14333

gse14333.expr <- read.delim(file.path(data.path,"gse14333.expr.txt"),sep = "\t",row.names = 1,check.names = F,stringsAsFactors = F,header = T)

gse14333.surv <- read.delim(file.path(data.path,"gse14333.surv.txt"),sep = "\t",row.names = 1,check.names = F,stringsAsFactors = F,header = T)

gse14333.surv$DFS.time <- as.numeric(gse14333.surv$DFS.time)

gse14333.surv$DFS <- as.numeric(gse14333.surv$DFS)

gse14333.surv <- gse14333.surv[!is.na(gse14333.surv$DFS),]

gse14333.expr <- gse14333.expr[,rownames(gse14333.surv)]

## gse17538

gse17538.expr <- read.delim(file.path(data.path,"gse17538.expr.txt"),sep = "\t",row.names = 1,check.names = F,stringsAsFactors = F,header = T)

gse17538.surv <- read.delim(file.path(data.path,"gse17538.surv.txt"),sep = "\t",row.names = 1,check.names = F,stringsAsFactors = F,header = T)

gse17538.surv <- gse17538.surv[!is.na(gse17538.surv$OS),]

gse17538.expr <- gse17538.expr[,rownames(gse17538.surv)]

## gse38832

gse38832.expr <- read.delim(file.path(data.path,"gse38832.expr.txt"),sep = "\t",row.names = 1,check.names = F,stringsAsFactors = F,header = T)

gse38832.surv <- read.delim(file.path(data.path,"gse38832.surv.txt"),sep = "\t",row.names = 1,check.names = F,stringsAsFactors = F,header = T)

## gse39582

gse39582.expr <- read.delim(file.path(data.path,"gse39582.expr.txt"),sep = "\t",row.names = 1,check.names = F,stringsAsFactors = F,header = T)

gse39582.surv <- read.delim(file.path(data.path,"gse39582.surv.txt"),sep = "\t",row.names = 1,check.names = F,stringsAsFactors = F,header = T)

gse39582.surv$OS <- as.numeric(gse39582.surv$OS)

gse39582.surv <- gse39582.surv[!is.na(gse39582.surv$OS),]

gse39582.surv$OS.time <- as.numeric(gse39582.surv$OS.time)

gse39582.expr <- gse39582.expr[,rownames(gse39582.surv)]

# combine geo cohorts

geo.expr <- cbind.data.frame(gse14333.expr, gse17538.expr,gse38832.expr, gse39582.expr)

geo.surv <- data.frame(OS.time = c(gse14333.surv$DFS.time, gse17538.surv$OS.time, gse38832.surv$OS.time, gse39582.surv$OS.time),

OS = c(gse14333.surv$DFS, gse17538.surv$OS, gse38832.surv$OS, gse39582.surv$OS),

Batch = rep(c("GSE14333","GSE17538","GSE38832","GSE39582"),c(ncol(gse14333.expr),ncol(gse17538.expr),ncol(gse38832.expr),ncol(gse39582.expr))),

row.names = c(colnames(gse14333.expr),colnames(gse17538.expr),colnames(gse38832.expr),colnames(gse39582.expr)))

modcombat = model.matrix(~1, data = geo.surv)

geo.expr.combat <- as.data.frame(ComBat(dat=as.matrix(geo.expr), batch=geo.surv$Batch, mod=modcombat))

write.table(geo.expr.combat, file.path(data.path,"geo.expr.combat.txt"),sep = "\t",row.names = T,col.names = NA,quote = F)

batchPCA(indata = t(scale(t(geo.expr.combat))),

batch = geo.surv$Batch,

fig.dir = fig.path,

PCA.fig.title = "Combat PCA for combined expression profile of Affymetrix",

cols = npg[1:4],

showID = F,

cex = 0.7,

showLegend = T)

batchPCA(indata = t(scale(t(geo.expr))),

batch = geo.surv$Batch,

fig.dir = fig.path,

PCA.fig.title = "Raw PCA for combined expression profile of Affymetrix",

cols = npg[1:4],

showID = F,

cex = 0.7,

showLegend = T)

# tide

geo.tide <- geo.expr.combat

geo.tide <- geo.tide[rowSums(geo.tide) > 0,]

geo.tide <- sweep(geo.tide[,rownames(annCol.geo)],1,apply(geo.tide, 1, median))

# geo.tide <- sweep(geo.tide,2, apply(geo.tide,2,median,na.rm=T))

# geo.tide <- round(sweep(geo.tide,1, apply(geo.tide,1,median,na.rm=T)),3)

write.table(geo.tide,file.path(res.path,"geo.tide.input.txt"),sep = "\t",row.names = T,col.names = NA,quote = F)

#--------------------------#

# validation in geo cohort #

mcp.geo <- MCPcounter.estimate(as.matrix(geo.expr.combat[,rownames(geo.surv)]),

featuresType = "HUGO_symbols",

genes = read.table(file.path(comAnn.path,"genes.txt"),sep = "\t",stringsAsFactors = F,header = T,colClasses = "character",check.names = F),

probesets = read.table(file.path(comAnn.path,"probesets.txt"),sep = "\t",stringsAsFactors = F,header = F,colClasses = "character",check.names = F))

indata <- geo.expr.combat[,rownames(geo.surv)]

write.table(indata,file = file.path(res.path,"GEO_combinedExpr_hugo.txt"),sep = "\t",row.names = T,col.names = NA,quote = F)

filterCommonGenes(input.f=file.path(res.path, "GEO_combinedExpr_hugo.txt") , output.f=file.path(res.path,"GEO_combinedExpr_hugo_ESTIMATE.txt"), id="GeneSymbol")

estimateScore(file.path(res.path,"GEO_combinedExpr_hugo_ESTIMATE.txt"), file.path(res.path,"GEO_combinedExpr_hugo_estimate_score.txt"), platform="affymetrix")

est.geo <- read.table(file = file.path(res.path,"GEO_combinedExpr_hugo_estimate_score.txt"),header = T,row.names = NULL,check.names = F,stringsAsFactors = F,sep = "\t")

rownames(est.geo) <- est.geo[,2]; colnames(est.geo) <- est.geo[1,]; est.geo <- est.geo[-1,c(-1,-2)];

est.geo <- sapply(est.geo, as.numeric); rownames(est.geo) <- c("StromalScore","ImmuneScore","ESTIMATEScore","TumorPurity"); est.geo.backup = as.data.frame(est.geo); colnames(est.geo.backup) <- colnames(indata)

est.geo <- annTrackScale(indata = est.geo, halfwidth = 2, poolsd = F); est.geo <- as.data.frame(t(est.geo))

rownames(est.geo) <- rownames(geo.surv)

annCol.geo <- geo.surv[,c("OS","Batch")]

annCol.geo <- annCol.geo[est.geo.backup[4,rownames(annCol.geo)] < 0.8,]

annCol.geo$IES <- est.geo[rownames(annCol.geo),"ImmuneScore"]

annCol.geo$SES <- est.geo[rownames(annCol.geo),"StromalScore"]

annColors.geo <- list()

annColors.geo[["IES"]] <- annColors$IES

annColors.geo[["SES"]] <- annColors$SES

annColors.geo[["Batch"]] <- c("GSE14333" = npg[1],"GSE17538" = npg[2],"GSE38832" = npg[3],"GSE39582" = npg[4])

tmp <- geo.expr.combat

immunosuppression.geoMean.geo <- apply(tmp[immunosuppression.signature,], 2, geoMean)

t.cell.activation.geoMean.geo <- apply(tmp[t.cell.activation.signature,], 2, geoMean)

t.cell.survival.geoMean.geo <- apply(tmp[t.cell.survival.signature,], 2, geoMean)

regulatory.t.cell.geoMean.geo <- apply(tmp[regulatory.t.cell.signature,], 2, geoMean)

mhc.geoMean.geo <- apply(tmp[mhc.signature,], 2, geoMean)

myeloid.geoMean.geo <- apply(tmp[myeloid.signature,], 2, geoMean)

tls.geoMean.geo <- apply(tmp[tls.signature,], 2, geoMean)

indata <- t(scale(t(mcp.geo[,rownames(annCol.geo)])))

hcs <- hclust(distanceMatrix(as.matrix(indata), "euclidean"), "ward.D2") # 原文提到使用欧式距离以及ward's聚类方式

group <- cutree(hcs, k = 4)

group <- paste0("CS", group); names(group) <- rownames(annCol.geo)

group <- sapply(group,function(x) {

switch(x,

"CS1" = "CS4",

"CS3" = "CS2",

"CS4" = "CS1",

"CS2" = "CS3")})

annCol.geo$ImmClust <- group[rownames(annCol.geo)]

annCol.geo <- annCol.geo[order(annCol.geo$ImmClust),]

annColors.geo[["ImmClust"]] <- c("CS1" = mycol[7],"CS2" = mycol[5],"CS3" = mycol[3],"CS4" = mycol[1])

annColors.geo[["OS"]] <- c("1" = "black","0" = "grey90")

geo.tide.res <- read.csv(file.path(res.path,"geo.tide.output.csv"),row.names = 1,check.names = F,stringsAsFactors = F,header = T)

geo.tide.res$ImmClust <- annCol.geo[rownames(geo.tide.res),"ImmClust"]

table(geo.tide.res$ImmClust, geo.tide.res$Responder)

boxplot(geo.tide.res$TIDE~geo.tide.res$ImmClust)

pairwise.wilcox.test(geo.tide.res$TIDE,geo.tide.res$ImmClust)

annCol.geo$Responder <- geo.tide.res[rownames(annCol.geo),"Responder"]

annCol.geo$TIDE <- geo.tide.res[rownames(annCol.geo),"TIDE"]

annColors.geo[["Responder"]] <- c("True" = cherry,"False" = "grey90")

annColors.geo[["TIDE"]] <- bluered(64)

#annCol.geo <- annCol.geo[order(annCol.geo$ImmClust),]

tme.dat.geo <- data.frame("Immunosuppression" = immunosuppression.geoMean.geo,

"T cell activation" = t.cell.activation.geoMean.geo,

"T cell survival" = t.cell.survival.geoMean.geo,

"Regulatory T cell" = regulatory.t.cell.geoMean.geo,

"Class I MHC" = mhc.geoMean.geo,

"Myeloid cells chemotactism" = myeloid.geoMean.geo,

"TLSs" = tls.geoMean.geo)

## MCPcounter heatmap

indata <- mcp.geo

plotdata <- standarize.fun(indata, halfwidth = 2)

hm1 <- pheatmap(plotdata[,rownames(annCol.geo)],

border_color = NA, # 无边框

color = bluered(64), # 采用红蓝颜色

show_rownames = T, # 显示行名

show_colnames = F, # 不显示列名

cluster_rows = F, # 行不聚类

cluster_cols = F, # 列不聚类

name = "Gene/metagene\nZ-score", # 颜色图例的名字（所有热图统一，这样只会显示一个）

cellheight = 12, # 热图元素高度

cellwidth = 300/ncol(plotdata), # 热图总宽度固定为300

annotation_col = annCol.geo[,c("ImmClust","Batch","IES","SES","TIDE","Responder")], # 样本注释

annotation_colors = annColors.geo[c("ImmClust","Batch","IES","SES","TIDE","Responder")]) # 注释颜色

## other signature heatmap

plotdata <- standarize.fun(t(tme.dat.geo[rownames(annCol.geo),]), halfwidth = 2)

hm2 <- pheatmap(plotdata,

border_color = NA,

color = bluered(64),

show_rownames = T,

show_colnames = F,

cluster_rows = F,

cluster_cols = F,

name = "Gene/metagene\nZ-score",

cellheight = 12,

cellwidth = 300/ncol(plotdata))

## ici heatmap

plotdata <- standarize.fun(geo.expr.combat[ici.gene,rownames(annCol.geo)], halfwidth = 2)

hm3 <- pheatmap(plotdata,

border_color = NA,

color = bluered(64),

show_rownames = T,

show_colnames = F,

cluster_rows = F,

cluster_cols = F,

name = "Gene/metagene\nZ-score",

cellheight = 12,

cellwidth = 300/ncol(plotdata))

pdf(file = file.path(fig.path,"tme heatmap of geo.pdf"), width = 10,height = 12)

draw(hm1 %v% hm2 %v% hm3, # 纵向合并三个热图

heatmap_legend_side = "bottom", # 热图图例显示在左侧

annotation_legend_side = "bottom") # 样本注释显示在左侧

invisible(dev.off())

fibgsva.geo <- gsva(as.matrix(geo.expr.combat),fibroblast.sig.cd,method = "gsva")

indata <- standarize.fun(geo.expr.combat[caf.gene, rownames(annCol.geo)], halfwidth = 2)

hm1 <- pheatmap(indata[,rownames(annCol.geo)],

border_color = NA, # 无边框

color = greenred(64), # 采用红蓝颜色

show_rownames = T, # 显示行名

show_colnames = F, # 不显示列名

cluster_rows = F, # 行不聚类

cluster_cols = F, # 列不聚类

name = "Gene\nZ-score", # 颜色图例的名字（所有热图统一，这样只会显示一个）

cellheight = 12, # 热图元素高度

cellwidth = 300/ncol(plotdata), # 热图总宽度固定为300

annotation_col = annCol.geo[,c("ImmClust","IES","SES","TIDE","Responder")], # 样本注释

annotation_colors = annColors.geo[c("ImmClust","IES","SES","TIDE","Responder")]) # 注释颜色

indata <- standarize.fun(fibgsva.geo, halfwidth = 1)

hm2 <- pheatmap(indata[,rownames(annCol.geo)],

border_color = NA, # 无边框

color = greenred(64), # 采用红蓝颜色

show_rownames = T, # 显示行名

show_colnames = F, # 不显示列名

cluster_rows = F, # 行不聚类

cluster_cols = F, # 列不聚类

name = "GSVA", # 颜色图例的名字（所有热图统一，这样只会显示一个）

cellheight = 12, # 热图元素高度

cellwidth = 300/ncol(plotdata)) # 注释颜色

pdf(file.path(fig.path,"heatmap of caf in geo.pdf"), width = 8, height = 8)

draw(hm1 %v% hm2, heatmap_legend_side = "bottom",annotation_legend_side = "bottom")

invisible(dev.off())

# submap

skcm.immunotherapy.logNC <- read.table(file.path(data.path,"skcm.immunotherapy.47samples.log2CountsNorm.txt"),sep = "\t",row.names = 1,header = T,check.names = F,stringsAsFactors = F) #鍘熸枃鎻愪緵鐨刲og2杞寲鐨勬爣鍑嗗寲count鍊?

rownames(skcm.immunotherapy.logNC) <- toupper(rownames(skcm.immunotherapy.logNC)) # 鍩哄洜澶у啓锛屽洜涓烘垜浣跨敤鐨勬暟鎹槸鎶婂熀鍥犲悕閮藉ぇ鍐欑殑

skcm.immunotherapy.info <- read.table(file.path(data.path,"skcm.immunotherapy.47sampleInfo.txt"),sep = "\t",row.names = 1,header = T,check.names = F,stringsAsFactors = F)

skcm.immunotherapy.info <- skcm.immunotherapy.info[order(skcm.immunotherapy.info$label),]

skcm.immunotherapy.info$rank <- rep(c(1,2,3,4),times=as.character(table(skcm.immunotherapy.info$label))) #1: CTLA4_noR 2: CTLA4_R 3:PD1_noR 4:PD1_R

sam_info <- skcm.immunotherapy.info

GENELIST <- intersect(rownames(geo.expr.combat),rownames(skcm.immunotherapy.logNC))

in_gct <- skcm.immunotherapy.logNC[GENELIST,rownames(skcm.immunotherapy.info)]

gct_file <- file.path(res.path,"skcm.immunotherapy.for.SubMap.gct")

cls_file <- file.path(res.path,"skcm.immunotherapy.for.SubMap.cls")

generateInputFileForSubMap(in_gct = in_gct, gct_file = gct_file, cls_file = cls_file, sam_info = sam_info, type_name = "rank")

# tcga

sam_info <- data.frame("ImmClust"=annCol.geo$ImmClust,row.names = rownames(annCol.geo))

sam_info$rank <- as.numeric(gsub("CS","",sam_info$ImmClust))

sam_info <- sam_info[order(sam_info$rank),]

gct_file <- file.path(res.path,"geo.immclust.for.SubMap.gct")

cls_file <- file.path(res.path,"geo.immclust.for.SubMap.cls")

in_gct <- geo.expr.combat[GENELIST,rownames(sam_info)]

generateInputFileForSubMap(in_gct = in_gct, gct_file = gct_file, cls_file = cls_file, sam_info = sam_info, type_name = "rank")

# survival analysis

tmp <- cbind.data.frame(geo.surv[rownames(annCol.geo),],

cluster = annCol.geo$ImmClust)

tmp$OS.time <- tmp$OS.time/30

fitd <- survdiff(Surv(OS.time, OS) ~ cluster, data=tmp, na.action=na.exclude)

p.val <- 1-pchisq(fitd$chisq, length(fitd$n)-1)

fit <- survfit(Surv(OS.time, OS)~ cluster, data=tmp, type="kaplan-meier", error="greenwood", conf.type="plain", na.action=na.exclude)

pairwise_survdiff(Surv(OS.time, OS)~ cluster,

data = tmp,

p.adjust.method = "none")

names(fit$strata) <- gsub("cluster=", "", names(fit$strata))

p <- ggsurvplot(fit, conf.int=F,risk.table=F, risk.table.col="strata",palette = mycol[c(7,5,3,1)],

pval = F,data=tmp,size=1,

tables.height = 0.3,surv.median.line = "hv",

xlab = "Time (Months)",ylab = "OS",

ylim = c(0,1),

risk.table.y.text = F)

p.lab <- paste0("Overall P",

ifelse(p.val < 0.001, " < 0.001",

paste0(" = ",round(p.val, 3))))

p$plot <- p$plot + annotate("text",

x = 0, y = 0.25,

hjust = 0,

fontface = 4,

label = p.lab)

pdf.options(reset = TRUE, onefile = FALSE)

pdf(file.path(fig.path,"km of os using immune cluster in coadread of geo.pdf"),width = 5,height = 5)

print(p)

dev.off()

# submap heatmap

tmp <- matrix(c(0.975,0.872,0.834,0.001, # nominal p value

0.823,0.116,0.893,0.001,

0.158,0.004,0.294,0.855,

0.236,0.644,0.381,0.991,

1,1,1,0.016, # Bonferroni adjusted p value

1,1,1,0.016,

1,0.064,1,1,

1,1,1,1,

1,1,1,0.011, # FDR adjusted p value

1,0.464,1,0.011,

0.505,0.021,0.671,1,

0.629,1,0.761,0.991),

nrow = 12,byrow = T,

dimnames = list(c("CS1","CS2","CS3","CS4"," CS1"," CS2"," CS3"," CS4"," CS1"," CS2"," CS3", " CS4"),c("CTAL4-noR","CTLA4-R","PD1-noR","PD1-R")))

hm <- pheatmap(t(tmp),

border_color = "white",

number_format = "%.3f",

cellwidth = 30, cellheight = 30,

cluster_rows = F,cluster_cols = F,

color = rev(NMF:::ccRamp(c("#E6EAF7","#B6D1E8","#498EB9","#204F8D"),64)),

display_numbers = T,

number_color = "black",

fontsize_number = 9,

name = "Statitic",

annotation_col = data.frame(pvalue=c("Nominal p value","Nominal p value","Nominal p value","Nominal p value","Bonferroni adjusted","Bonferroni adjusted","Bonferroni adjusted","Bonferroni adjusted","FDR adjusted","FDR adjusted","FDR adjusted","FDR adjusted"),

row.names = rownames(tmp)),

annotation_colors = list(pvalue=c("Nominal p value"="black","Bonferroni adjusted"="grey50","FDR adjusted" = "grey80")))

pdf(file.path(fig.path,"submap heatmap of predicted response to immunotherapy in geo coadread.pdf"),width = 10,height = 4)

draw(hm, heatmap_legend_side = "left",annotation_legend_side = "bottom")

invisible(dev.off())

#--------------------------------#

# copy number variation analysis #

cnv <- read.table(file.path(data.path,"COADREAD.snp__genome_wide_snp_6__broad_mit_edu__Level_3__segmented_scna_minus_germline_cnv_hg19__seg.seg.txt"),sep = "\t",row.names = NULL,header = T,check.names = F,stringsAsFactors = F)

cnv$Sample <-substr(cnv$Sample,start = 1,stop = 16)

comsam <- intersect(cnv$Sample,rownames(annCol)) # 520

cnv <- cnv[which(cnv$Sample %in% comsam),]

write.table(as.data.frame(cnv),file.path(res.path,"coadread_520_segment_forGISTIC2.0.txt"),sep = "\t",row.names = F,quote = F)

# create marker file for GISTIC2.0

marker <- cnv[,1:4]

a <- b <- c <- c()

for(i in 1:nrow(marker)) {

a <- c(a,rep(marker[i,"Sample"],2))

b <- c(b,rep(marker[i,"Chromosome"],2))

c <- c(c,marker[i,"Start"],marker[i,"End"])

}

marker <- data.frame(Marker.Name = a,Chromosome = b,Marker.Position=c,stringsAsFactors = F)

write.table(as.data.frame(marker),file.path(res.path,"coadread_520_marker_forGISTIC2.0.txt"),sep = "\t",row.names = F,quote = F)

rm(a); rm(b); rm(c)

# seperate perform GISTIC2.0

seg.C1.cnv <- cnv[which(cnv$Sample %in% rownames(annCol[which(annCol$ImmClust == "CS1"),])),]

seg.C2.cnv <- cnv[which(cnv$Sample %in% rownames(annCol[which(annCol$ImmClust == "CS2"),])),]

seg.C3.cnv <- cnv[which(cnv$Sample %in% rownames(annCol[which(annCol$ImmClust == "CS3"),])),]

seg.C4.cnv <- cnv[which(cnv$Sample %in% rownames(annCol[which(annCol$ImmClust == "CS4"),])),]

write.table(as.data.frame(seg.C1.cnv),file.path(res.path,"coadread_cs1_segment_forGISTIC2.0.txt"),sep = "\t",row.names = F,quote = F)

write.table(as.data.frame(seg.C2.cnv),file.path(res.path,"coadread_cs2_segment_forGISTIC2.0.txt"),sep = "\t",row.names = F,quote = F)

write.table(as.data.frame(seg.C3.cnv),file.path(res.path,"coadread_cs3_segment_forGISTIC2.0.txt"),sep = "\t",row.names = F,quote = F)

write.table(as.data.frame(seg.C4.cnv),file.path(res.path,"coadread_cs4_segment_forGISTIC2.0.txt"),sep = "\t",row.names = F,quote = F)

# create marker file for GISTIC2.0

marker <- seg.C1.cnv[,1:4]

a <- b <- c <- c()

for(i in 1:nrow(marker)) {

a <- c(a,rep(marker[i,"Sample"],2))

b <- c(b,rep(marker[i,"Chromosome"],2))

c <- c(c,marker[i,"Start"],marker[i,"End"])

}

marker <- data.frame(Marker.Name = a,Chromosome = b,Marker.Position=c,stringsAsFactors = F)

write.table(as.data.frame(marker),file.path(res.path,"coadread_cs1_marker_forGISTIC2.0.txt"),sep = "\t",row.names = F,quote = F)

rm(a); rm(b); rm(c)

marker <- seg.C2.cnv[,1:4]

a <- b <- c <- c()

for(i in 1:nrow(marker)) {

a <- c(a,rep(marker[i,"Sample"],2))

b <- c(b,rep(marker[i,"Chromosome"],2))

c <- c(c,marker[i,"Start"],marker[i,"End"])

}

marker <- data.frame(Marker.Name = a,Chromosome = b,Marker.Position=c,stringsAsFactors = F)

write.table(as.data.frame(marker),file.path(res.path,"coadread_cs2_marker_forGISTIC2.0.txt"),sep = "\t",row.names = F,quote = F)

rm(a); rm(b); rm(c)

marker <- seg.C3.cnv[,1:4]

a <- b <- c <- c()

for(i in 1:nrow(marker)) {

a <- c(a,rep(marker[i,"Sample"],2))

b <- c(b,rep(marker[i,"Chromosome"],2))

c <- c(c,marker[i,"Start"],marker[i,"End"])

}

marker <- data.frame(Marker.Name = a,Chromosome = b,Marker.Position=c,stringsAsFactors = F)

write.table(as.data.frame(marker),file.path(res.path,"coadread_cs3_marker_forGISTIC2.0.txt"),sep = "\t",row.names = F,quote = F)

rm(a); rm(b); rm(c)

marker <- seg.C4.cnv[,1:4]

a <- b <- c <- c()

for(i in 1:nrow(marker)) {

a <- c(a,rep(marker[i,"Sample"],2))

b <- c(b,rep(marker[i,"Chromosome"],2))

c <- c(c,marker[i,"Start"],marker[i,"End"])

}

marker <- data.frame(Marker.Name = a,Chromosome = b,Marker.Position=c,stringsAsFactors = F)

write.table(as.data.frame(marker),file.path(res.path,"coadread_cs4_marker_forGISTIC2.0.txt"),sep = "\t",row.names = F,quote = F)

rm(a); rm(b); rm(c)

# generate gistic results

# Create a chromosomes reference objects function

chrom_extract <- function(BSgenome.hg = NULL) {

if (is.null(BSgenome.hg )) stop("NULL object !", call. = FALSE)

obj <- list(species = GenomeInfoDb::organism(BSgenome.hg), genomebuild = BSgenome::providerVersion(BSgenome.hg))

df <- data.frame(chrom = BSgenome::seqnames(BSgenome.hg), chrN = seq_along(BSgenome::seqnames(BSgenome.hg)), chr.length = GenomeInfoDb::seqlengths(BSgenome.hg), stringsAsFactors = FALSE)

df <- df[1:24,]

df$chr.length.sum <- cumsum(as.numeric(df$chr.length))

df$chr.length.cumsum <- c(0, df$chr.length.sum[-nrow(df)])

df$middle.chr <- round(diff(c(0, df$chr.length.sum)) /2)

df$middle.chr.genome <- df$middle.chr + df$chr.length.cumsum

obj$chromosomes <- df

obj$chrom2chr <- sapply(obj$chromosomes$chrom, function(k) { obj$chromosomes$chrN[obj$chromosomes$chrom == k]}, simplify = FALSE)

obj$chr2chrom <- sapply(obj$chromosomes$chrN, function(k) { obj$chromosomes$chrom[obj$chromosomes$chrN == k]}, simplify = FALSE)

names(obj$chr2chrom) <- obj$chromosomes$chrN

obj$genome.length <- sum(as.numeric(obj$chromosomes$chr.length), na.rm = TRUE)

return(obj)

}

# Extract a chromosomes reference loci

BSgenome.hg = "BSgenome.Hsapiens.UCSC.hg19"

BSg.obj <- getExportedValue(BSgenome.hg, BSgenome.hg)

genome.version <- BSgenome::providerVersion(BSg.obj)

chrom <- chrom_extract(BSg.obj)

#str(chrom)

pdf(file = file.path(fig.path,"cnv scores from gistic in tcga coadread.pdf"),12,10)

par(mfrow=c(5,1), mgp = c(1.5,.33,0), mar=c(4,4,3,0.1), las=1, tcl=-.25,las = 1)

### all COADREAD ###

scores <- read.table(file.path(res.path,"GISTIC/ALL/coadread_520.scores.gistic"), sep="\t",header=T,stringsAsFactors = F)

# Important step for accurate length to match back to continual chrom loci

scores[scores$Chromosome==23,"Chromosome"]="X"

scores[scores$Chromosome==24,"Chromosome"]="Y"

chrID <- unname(unlist(chrom$chrom2chr[as.character(paste0("chr",scores$Chromosome))]))

scores$Start.geno <- scores$Start + chrom$chromosomes$chr.length.cumsum[chrID]

scores$End.geno <- scores$End + chrom$chromosomes$chr.length.cumsum[chrID]

# Prepare input data for ploting

scores.amp <- scores[scores$Type=="Amp",]

scores.amp$G.score <- scores.amp$G.score * 1

scores.del <- scores[scores$Type=="Del",]

scores.del$G.score <- scores.del$G.score * -1

scores <- rbind.data.frame(scores.amp,scores.del)

# seg.col = list(gain = "red", outscale.gain = "firebrick1", loss = "blue", outscale.red = "steelblue")

ylim <- c(min(scores$G.score)-0.1,max(scores$G.score)+0.1)

title="CN gistic score of all CRC cases (n = 520)"

plot(scores.amp$Start.geno, scores.amp$G.score,

pch = ".", type='h',cex = 1.5, xaxs = "i", yaxs = "i", xlim = c(0,chrom$genome.length), ylim = ylim,

main = title, cex.main = 1.5, ylab = "gistic score", xlab = NA,

cex.lab = 1.5, col = adjustcolor("firebrick1", alpha.f = .8), xaxt = "n", lwd = 2, las=1) # las=1 rotating axis labels in R

lines(scores.del$Start.geno, scores.del$G.score, type='h', lwd = 2, col = adjustcolor("steelblue", alpha.f = .8))

ink <- chrom$chromosomes$chrN %in% chrID

yrange = abs(diff(ylim))

m.pos <- c(ylim[1]+0.05,ylim[2]-0.05)

m.mod <- -(chrom$chromosomes$chrN[ink] %% 2) +2

try(text(x = chrom$chromosomes$middle.chr.geno[ink], y = m.pos[m.mod], labels = chrom$chromosomes$chrom[ink], cex = 1))

abline(h = 0.0, col = 1, lwd = 1, lty = 3)

abline(v = c(0,chrom$chromosomes$chr.length.sum), col = 1, lty = 3, lwd = 1)

col1 <- adjustcolor("firebrick1", alpha.f = .8)

col2 <- adjustcolor("steelblue", alpha.f = .8)

# The position of the legend can be specified also using the following keywords : "bottomright", "bottom", "bottomleft", "left", "topleft", "top", "topright", "right" and "center".

legend("topleft", c("gain","loss"), cex=0.6, bty="n", fill=c(col1,col2))

### CS1 ###

scores <- read.table(file.path(res.path,"GISTIC/CS1/coadread_cs1.scores.gistic"), sep="\t",header=T,stringsAsFactors = F)

# Important step for accurate length to match back to continual chrom loci

scores[scores$Chromosome==23,"Chromosome"]="X"

scores[scores$Chromosome==24,"Chromosome"]="Y"

chrID <- unname(unlist(chrom$chrom2chr[as.character(paste0("chr",scores$Chromosome))]))

scores$Start.geno <- scores$Start + chrom$chromosomes$chr.length.cumsum[chrID]

scores$End.geno <- scores$End + chrom$chromosomes$chr.length.cumsum[chrID]

# Prepare input data for ploting

scores.amp <- scores[scores$Type=="Amp",]

scores.amp$G.score <- scores.amp$G.score * 1

scores.del <- scores[scores$Type=="Del",]

scores.del$G.score <- scores.del$G.score * -1

scores <- rbind.data.frame(scores.amp,scores.del)

# seg.col = list(gain = "red", outscale.gain = "firebrick1", loss = "blue", outscale.red = "steelblue")

ylim <- c(min(scores$G.score)-0.1,max(scores$G.score)+0.1)

title="CN gistic score of ImmClust CS1 (n = 83)"

plot(scores.amp$Start.geno, scores.amp$G.score,

pch = ".", type='h',cex = 1.5, xaxs = "i", yaxs = "i", xlim = c(0,chrom$genome.length), ylim = ylim,

main = title, cex.main = 1.5, ylab = "gistic score", xlab = NA,

cex.lab = 1.5, col = adjustcolor("firebrick1", alpha.f = .8), xaxt = "n", lwd = 2, las=1) # las=1 rotating axis labels in R

lines(scores.del$Start.geno, scores.del$G.score, type='h', lwd = 2, col = adjustcolor("steelblue", alpha.f = .8))

ink <- chrom$chromosomes$chrN %in% chrID

yrange = abs(diff(ylim))

m.pos <- c(ylim[1]+0.05,ylim[2]-0.05)

m.mod <- -(chrom$chromosomes$chrN[ink] %% 2) +2

try(text(x = chrom$chromosomes$middle.chr.geno[ink], y = m.pos[m.mod], labels = chrom$chromosomes$chrom[ink], cex = 1))

abline(h = 0.0, col = 1, lwd = 1, lty = 3)

abline(v = c(0,chrom$chromosomes$chr.length.sum), col = 1, lty = 3, lwd = 1)

col1 <- adjustcolor("firebrick1", alpha.f = .8)

col2 <- adjustcolor("steelblue", alpha.f = .8)

# The position of the legend can be specified also using the following keywords : "bottomright", "bottom", "bottomleft", "left", "topleft", "top", "topright", "right" and "center".

legend("topleft", c("gain","loss"), cex=0.6, bty="n", fill=c(col1,col2))

### CS2 ###

scores <- read.table(file.path(res.path,"GISTIC/CS2/coadread_cs2.scores.gistic"), sep="\t",header=T,stringsAsFactors = F)

# Important step for accurate length to match back to continual chrom loci

scores[scores$Chromosome==23,"Chromosome"]="X"

scores[scores$Chromosome==24,"Chromosome"]="Y"

chrID <- unname(unlist(chrom$chrom2chr[as.character(paste0("chr",scores$Chromosome))]))

scores$Start.geno <- scores$Start + chrom$chromosomes$chr.length.cumsum[chrID]

scores$End.geno <- scores$End + chrom$chromosomes$chr.length.cumsum[chrID]

# Prepare input data for ploting

scores.amp <- scores[scores$Type=="Amp",]

scores.amp$G.score <- scores.amp$G.score * 1

scores.del <- scores[scores$Type=="Del",]

scores.del$G.score <- scores.del$G.score * -1

scores <- rbind.data.frame(scores.amp,scores.del)

# seg.col = list(gain = "red", outscale.gain = "firebrick1", loss = "blue", outscale.red = "steelblue")

ylim <- c(min(scores$G.score)-0.1,max(scores$G.score)+0.1)

title="CN gistic score of ImmClust CS2 (n = 75)"

plot(scores.amp$Start.geno, scores.amp$G.score,

pch = ".", type='h',cex = 1.5, xaxs = "i", yaxs = "i", xlim = c(0,chrom$genome.length), ylim = ylim,

main = title, cex.main = 1.5, ylab = "gistic score", xlab = NA,

cex.lab = 1.5, col = adjustcolor("firebrick1", alpha.f = .8), xaxt = "n", lwd = 2, las=1) # las=1 rotating axis labels in R

lines(scores.del$Start.geno, scores.del$G.score, type='h', lwd = 2, col = adjustcolor("steelblue", alpha.f = .8))

ink <- chrom$chromosomes$chrN %in% chrID

yrange = abs(diff(ylim))

m.pos <- c(ylim[1]+0.05,ylim[2]-0.05)

m.mod <- -(chrom$chromosomes$chrN[ink] %% 2) +2

try(text(x = chrom$chromosomes$middle.chr.geno[ink], y = m.pos[m.mod], labels = chrom$chromosomes$chrom[ink], cex = 1))

abline(h = 0.0, col = 1, lwd = 1, lty = 3)

abline(v = c(0,chrom$chromosomes$chr.length.sum), col = 1, lty = 3, lwd = 1)

col1 <- adjustcolor("firebrick1", alpha.f = .8)

col2 <- adjustcolor("steelblue", alpha.f = .8)

# The position of the legend can be specified also using the following keywords : "bottomright", "bottom", "bottomleft", "left", "topleft", "top", "topright", "right" and "center".

legend("topleft", c("gain","loss"), cex=0.6, bty="n", fill=c(col1,col2))

### CS3 ###

scores <- read.table(file.path(res.path,"GISTIC/CS3/coadread_cs3.scores.gistic"), sep="\t",header=T,stringsAsFactors = F)

# Important step for accurate length to match back to continual chrom loci

scores[scores$Chromosome==23,"Chromosome"]="X"

scores[scores$Chromosome==24,"Chromosome"]="Y"

chrID <- unname(unlist(chrom$chrom2chr[as.character(paste0("chr",scores$Chromosome))]))

scores$Start.geno <- scores$Start + chrom$chromosomes$chr.length.cumsum[chrID]

scores$End.geno <- scores$End + chrom$chromosomes$chr.length.cumsum[chrID]

# Prepare input data for ploting

scores.amp <- scores[scores$Type=="Amp",]

scores.amp$G.score <- scores.amp$G.score * 1

scores.del <- scores[scores$Type=="Del",]

scores.del$G.score <- scores.del$G.score * -1

scores <- rbind.data.frame(scores.amp,scores.del)

# seg.col = list(gain = "red", outscale.gain = "firebrick1", loss = "blue", outscale.red = "steelblue")

ylim <- c(min(scores$G.score)-0.1,max(scores$G.score)+0.1)

title="CN gistic score of ImmClust CS3 (n = 124)"

plot(scores.amp$Start.geno, scores.amp$G.score,

pch = ".", type='h',cex = 1.5, xaxs = "i", yaxs = "i", xlim = c(0,chrom$genome.length), ylim = ylim,

main = title, cex.main = 1.5, ylab = "gistic score", xlab = NA,

cex.lab = 1.5, col = adjustcolor("firebrick1", alpha.f = .8), xaxt = "n", lwd = 2, las=1) # las=1 rotating axis labels in R

lines(scores.del$Start.geno, scores.del$G.score, type='h', lwd = 2, col = adjustcolor("steelblue", alpha.f = .8))

ink <- chrom$chromosomes$chrN %in% chrID

yrange = abs(diff(ylim))

m.pos <- c(ylim[1]+0.05,ylim[2]-0.05)

m.mod <- -(chrom$chromosomes$chrN[ink] %% 2) +2

try(text(x = chrom$chromosomes$middle.chr.geno[ink], y = m.pos[m.mod], labels = chrom$chromosomes$chrom[ink], cex = 1))

abline(h = 0.0, col = 1, lwd = 1, lty = 3)

abline(v = c(0,chrom$chromosomes$chr.length.sum), col = 1, lty = 3, lwd = 1)

col1 <- adjustcolor("firebrick1", alpha.f = .8)

col2 <- adjustcolor("steelblue", alpha.f = .8)

# The position of the legend can be specified also using the following keywords : "bottomright", "bottom", "bottomleft", "left", "topleft", "top", "topright", "right" and "center".

legend("topleft", c("gain","loss"), cex=0.6, bty="n", fill=c(col1,col2))

### CS4 ###

scores <- read.table(file.path(res.path,"GISTIC/CS4/coadread_cs4.scores.gistic"), sep="\t",header=T,stringsAsFactors = F)

# Important step for accurate length to match back to continual chrom loci

scores[scores$Chromosome==23,"Chromosome"]="X"

scores[scores$Chromosome==24,"Chromosome"]="Y"

chrID <- unname(unlist(chrom$chrom2chr[as.character(paste0("chr",scores$Chromosome))]))

scores$Start.geno <- scores$Start + chrom$chromosomes$chr.length.cumsum[chrID]

scores$End.geno <- scores$End + chrom$chromosomes$chr.length.cumsum[chrID]

# Prepare input data for ploting

scores.amp <- scores[scores$Type=="Amp",]

scores.amp$G.score <- scores.amp$G.score * 1

scores.del <- scores[scores$Type=="Del",]

scores.del$G.score <- scores.del$G.score * -1

scores <- rbind.data.frame(scores.amp,scores.del)

# seg.col = list(gain = "red", outscale.gain = "firebrick1", loss = "blue", outscale.red = "steelblue")

ylim <- c(min(scores$G.score)-0.1,max(scores$G.score)+0.1)

title="CN gistic score of ImmClust CS4 (n = 238)"

plot(scores.amp$Start.geno, scores.amp$G.score,

pch = ".", type='h',cex = 1.5, xaxs = "i", yaxs = "i", xlim = c(0,chrom$genome.length), ylim = ylim,

main = title, cex.main = 1.5, ylab = "gistic score", xlab = NA,

cex.lab = 1.5, col = adjustcolor("firebrick1", alpha.f = .8), xaxt = "n", lwd = 2, las=1) # las=1 rotating axis labels in R

lines(scores.del$Start.geno, scores.del$G.score, type='h', lwd = 2, col = adjustcolor("steelblue", alpha.f = .8))

ink <- chrom$chromosomes$chrN %in% chrID

yrange = abs(diff(ylim))

m.pos <- c(ylim[1]+0.05,ylim[2]-0.05)

m.mod <- -(chrom$chromosomes$chrN[ink] %% 2) +2

try(text(x = chrom$chromosomes$middle.chr.geno[ink], y = m.pos[m.mod], labels = chrom$chromosomes$chrom[ink], cex = 1))

abline(h = 0.0, col = 1, lwd = 1, lty = 3)

abline(v = c(0,chrom$chromosomes$chr.length.sum), col = 1, lty = 3, lwd = 1)

col1 <- adjustcolor("firebrick1", alpha.f = .8)

col2 <- adjustcolor("steelblue", alpha.f = .8)

# The position of the legend can be specified also using the following keywords : "bottomright", "bottom", "bottomleft", "left", "topleft", "top", "topright", "right" and "center".

legend("topleft", c("gain","loss"), cex=0.6, bty="n", fill=c(col1,col2))

dev.off()

all.lesions <- file.path(res.path, "GISTIC/All/coadread_520.all_lesions.conf_95.txt")

amp.genes <- file.path(res.path, "GISTIC/All/coadread_520.amp_genes.conf_95.txt")

del.genes <- file.path(res.path, "GISTIC/All/coadread_520.del_genes.conf_95.txt")

scores.gis <- file.path(res.path, "GISTIC/All/coadread_520.scores.gistic")

gistic <- readGistic(gisticAllLesionsFile = all.lesions, gisticAmpGenesFile = amp.genes, gisticDelGenesFile = del.genes, gisticScoresFile = scores.gis, isTCGA = F)

cnvSummary <- as.data.frame(gistic@cnv.summary)

rownames(cnvSummary) <- cnvSummary$Tumor_Sample_Barcode

comsam <- intersect(rownames(annCol),rownames(cnvSummary))

cnvSummary[comsam,"ImmClust"] <- annCol[comsam,"ImmClust"]

# generate boxplot

ggplot(data = cnvSummary,aes(x = ImmClust,

y = log10(Amp + 1),

fill = ImmClust))+

scale_fill_manual(values = mycol[c(7,5,3,1)]) +

geom_violin(alpha = 0.4, position = position_dodge(width = .75),

size = 0.8, color="black") +

geom_boxplot(notch = TRUE, outlier.size = -1,

color="black", lwd=0.8, alpha = 0.7) +

geom_point(shape = 21, size=2,

position = position_jitterdodge(),

color="black", alpha = 1) +

theme_bw() +

ylab("log10(copy number amplification)") +

xlab("") +

theme(axis.text.x = element_text(hjust = 1, size = 12, color = "black"),

axis.ticks = element_line(size=0.2, color="black"),

axis.ticks.length = unit(0.2, "cm"),

legend.position = "top",

panel.background = element_blank(),

panel.grid = element_blank(),

axis.title = element_text(size = 12),

axis.text = element_text(size = 12)) +

stat_compare_means(method = "kruskal.test", label.y = min(log10(cnvSummary$Amp + 1))-0.3)

ggsave(file.path(fig.path,"boxplot for amplification in immclust of tcga.pdf"),width = 4,height = 5)

ggplot(data = cnvSummary,aes(x = ImmClust,

y = log10(Del + 1),

fill = ImmClust))+

scale_fill_manual(values = mycol[c(7,5,3,1)]) +

geom_violin(alpha = 0.4, position = position_dodge(width = .75),

size = 0.8, color="black") +

geom_boxplot(notch = TRUE, outlier.size = -1,

color="black", lwd=0.8, alpha = 0.7) +

geom_point(shape = 21, size=2,

position = position_jitterdodge(),

color="black", alpha = 1) +

theme_bw() +

ylab("log10(copy number deletion)") +

xlab("") +

theme(axis.text.x = element_text(hjust = 1, size = 12, color = "black"),

axis.ticks = element_line(size=0.2, color="black"),

axis.ticks.length = unit(0.2, "cm"),

legend.position = "top",

panel.background = element_blank(),

panel.grid = element_blank(),

axis.title = element_text(size = 12),

axis.text = element_text(size = 12)) +

stat_compare_means(method = "kruskal.test", label.y = min(log10(cnvSummary$Del + 1))-0.3)

ggsave(file.path(fig.path,"boxplot for deletion in immclust of tcga.pdf"),width = 4,height = 5)

#-------------------------------------------#

# use MOVICS to characterize three subtypes #

## mutational frequency

coadread.movics <- list("clust.res" = annCol,

"mo.method" = "coadread.icluster")

coadread.movics$clust.res$samID <- rownames(coadread.movics$clust.res)

coadread.movics$clust.res$clust <- sapply(coadread.movics$clust.res$ImmClust,

switch,

"CS1" = 1,

"CS2" = 2,

"CS3" = 3,

"CS4" = 4)

coadread.movics$clust.res <- coadread.movics$clust.res[intersect(rownames(coadread.movics$clust.res),colnames(mut.binary)),]

mut.immclust <- compMut(moic.res = coadread.movics,

mut.matrix = mut.binary, # binary somatic mutation matrix

clust.col = mycol[c(7,5,3,1)],

doWord = TRUE, # generate table in .docx format

doPlot = TRUE, # draw OncoPrint

freq.cutoff = 0.1, # keep those genes that mutated in at least 10% of samples

p.cutoff = 0.05, # keep those genes with nominal p value < 0.05 to draw OncoPrint

p.adj.cutoff = 0.05, # keep those genes with adjusted p value < 0.05 to draw OncoPrint

innerclust = T, # perform clustering within each subtype

annCol = annCol[colnames(mut.binary),"Responder",drop = F], # same annotation for heatmap

annColors = annColors[c("Responder")], # same annotation color for heatmap

width = 8,

height = 3.5,

fig.name = "oncoprint for movics siginificant mutations",

tab.name = "independent test between immclust and mutation",

res.path = res.path,

fig.path = fig.path)

# load driver mutation

driverMut <- read.delim(file.path(comAnn.path,"driver mutation consensus list.txt"),sep = "\t",row.names = NULL,check.names = F,stringsAsFactors = F,header = T)

driverMut <- driverMut[which(driverMut$Cancer == "COADREAD"),"Gene"]

## TMB

maf.coadread <- read_tsv(file.path(data.path,"TCGA_COADREAD_MAF.txt"), comment = "#")

maf.coadread$Tumor_Sample_Barcode <- paste0(maf.coadread$Tumor_Sample_Barcode,"A")

tmb.immclust <- compTMB(moic.res = coadread.movics,

maf = maf.coadread,

clust.col = mycol[c(7,5,3,1)],

rmDup = TRUE, # remove duplicated variants per sample

rmFLAGS = FALSE, # keep FLAGS mutations

exome.size = 38, # estimated exome size

test.method = "nonparametric", # statistical testing method

fig.path = fig.path,

fig.name = "distribution of TMB and TiTv")

# CS1 CS2 CS3

# CS2 "6.17e-01" " NA" " NA"

# CS3 "1.51e-02" "2.10e-02" " NA"

# CS4 "3.60e-01" "6.17e-01" "1.51e-02"

# oncoprint

mut.sig <- mut.immclust[which(as.numeric(mut.immclust$pvalue) < 0.05 & as.numeric(mut.immclust$padj) < 0.05),]

mut.sig <- mut.sig$`Gene (Mutated)`

maf_subset <- as.data.frame(maf[,c("Tumor_Sample_Barcode","Hugo_Symbol","Variant_Classification")])

onco.input <- reshape(data = maf_subset,idvar = "Hugo_Symbol",timevar = "Tumor_Sample_Barcode",direction = "wide")

onco.input <- onco.input[which(onco.input$Hugo_Symbol %in% mut.sig),]

colnames(onco.input) <- gsub("Variant_Classification.","",colnames(onco.input))

onco.input[is.na(onco.input)] <- ""

rownames(onco.input) <- onco.input$Hugo_Symbol

onco.input[onco.input == "5'Flank"] <- "Flank5"

onco.input[onco.input == "3'Flank"] <- "Flank3"

onco.input[onco.input == "5'UTR"] <- "UTR5"

onco.input[onco.input == "3'UTR"] <- "UTR3"

onco.input <- onco.input[,-1]

colnames(onco.input) <- paste0(colnames(onco.input),"A")

write.table(onco.input,file.path(res.path,"coadread differential mutated gene matrix in wide format.txt"),sep = "\t",row.names = T,col.names = NA,quote = F)

mut.col <- c(brewer.pal(12, "Paired"),"grey80")

type <- c()

for (i in 1:nrow(onco.input)) {

tmp <- as.character(onco.input[i,])

type <- unique(c(type,tmp))

}

# [1] "Missense_Mutation" "" "Silent" "In_Frame_Del"

# [5] "Nonsense_Mutation" "UTR3" "Frame_Shift_Del" "Frame_Shift_Ins"

# [9] "Flank5" "Splice_Site" "UTR5" "Intron"

# [13] "Splice_Region" "In_Frame_Ins"

alter_fun = list(

background = function(x, y, w, h) {

grid.rect(x, y, w-unit(0.5, "mm"), h-unit(0.5, "mm"), gp = gpar(fill = "#dcddde", col = NA))

},

Missense_Mutation = function(x, y, w, h) {

grid.rect(x, y, w-unit(0.5, "mm"), h-unit(0.5, "mm"), gp = gpar(fill = mut.col[1], col = NA))

},

Nonsense_Mutation = function(x, y, w, h) {

grid.rect(x, y, w-unit(0.5, "mm"), h-unit(0.5, "mm"), gp = gpar(fill = mut.col[2], col = NA))

},

Frame_Shift_Del = function(x, y, w, h) {

grid.rect(x, y, w-unit(0.5, "mm"), h-unit(0.5, "mm"), gp = gpar(fill = mut.col[3], col = NA))

},

Frame_Shift_Ins = function(x, y, w, h) {

grid.rect(x, y, w-unit(0.5, "mm"), h-unit(0.5, "mm"), gp = gpar(fill = mut.col[4], col = NA))

},

In_Frame_Del = function(x, y, w, h) {

grid.rect(x, y, w-unit(0.5, "mm"), h-unit(0.5, "mm"), gp = gpar(fill = mut.col[5], col = NA))

},

In_Frame_Ins = function(x, y, w, h) {

grid.rect(x, y, w-unit(0.5, "mm"), h-unit(0.5, "mm"), gp = gpar(fill = mut.col[6], col = NA))

},

Splice_Region = function(x, y, w, h) {

grid.rect(x, y, w-unit(0.5, "mm"), h-unit(0.5, "mm"), gp = gpar(fill = mut.col[7], col = NA))

},

Splice_Site = function(x, y, w, h) {

grid.rect(x, y, w-unit(0.5, "mm"), h-unit(0.5, "mm"), gp = gpar(fill = mut.col[8], col = NA))

},

UTR3 = function(x, y, w, h) {

grid.rect(x, y, w-unit(0.5, "mm"), h-unit(0.5, "mm"), gp = gpar(fill = mut.col[9], col = NA))

},

UTR5 = function(x, y, w, h) {

grid.rect(x, y, w-unit(0.5, "mm"), h-unit(0.5, "mm"), gp = gpar(fill = mut.col[10], col = NA))

},

Flank5 = function(x, y, w, h) {

grid.rect(x, y, w-unit(0.5, "mm"), h-unit(0.5, "mm"), gp = gpar(fill = mut.col[11], col = NA))

},

Intron = function(x, y, w, h) {

grid.rect(x, y, w-unit(0.5, "mm"), h-unit(0.5, "mm"), gp = gpar(fill = mut.col[12], col = NA))

},

Silent = function(x, y, w, h) {

grid.rect(x, y, w-unit(0.5, "mm"), h-unit(0.5, "mm"), gp = gpar(fill = mut.col[13], col = NA))

}

)

col = c("Missense_Mutation" = mut.col[1],

"Nonsense_Mutation" = mut.col[2],

"Frame_Shift_Del" = mut.col[3],

"Frame_Shift_Ins" = mut.col[4],

"In_Frame_Del" = mut.col[5],

"In_Frame_Ins" = mut.col[6],

"Splice_Region" = mut.col[7],

"Splice_Site" = mut.col[8],

"UTR3" = mut.col[9],

"UTR5" = mut.col[10],

"Flank5" = mut.col[11],

"Intron" = mut.col[12],

"Silent" = mut.col[13])

sample.C1 <- intersect(rownames(annCol[annCol$ImmClust == "CS1",]), colnames(onco.input))

sample.C2 <- intersect(rownames(annCol[annCol$ImmClust == "CS2",]), colnames(onco.input))

sample.C3 <- intersect(rownames(annCol[annCol$ImmClust == "CS3",]), colnames(onco.input))

sample.C4 <- intersect(rownames(annCol[annCol$ImmClust == "CS4",]), colnames(onco.input))

# do oncoprint seperately to get clustered sample order in suboncoprint

mut.order <- names(sort(rowSums(onco.input != ""),decreasing = T)) # get mutation order according to mutation frequency

p1 <- oncoPrint(onco.input[,sample.C1], # cluster1 oncoprint

alter_fun = alter_fun,

col = col,

row_order = mut.order)

p2 <- oncoPrint(onco.input[,sample.C2], # cluster2 oncoprint

alter_fun = alter_fun,

col = col,

row_order = mut.order)

p3 <- oncoPrint(onco.input[,sample.C3], # cluster3 oncoprint

alter_fun = alter_fun,

col = col,

row_order = mut.order)

p4 <- oncoPrint(onco.input[,sample.C4], # cluster4 oncoprint

alter_fun = alter_fun,

col = col,

row_order = mut.order)

my_ann <- data.frame(ImmClust = rep(c("CS1","CS2","CS3","CS4"),

c(length(sample.C1),length(sample.C2),length(sample.C3),length(sample.C4))),

row.names = c(sample.C1,sample.C2,sample.C3,sample.C4))

my_ann <- my_ann[c(sample.C1[p1@column_order],sample.C2[p2@column_order],sample.C3[p3@column_order],sample.C4[p4@column_order]),,drop = F] # sort sample by the suboncoprint order

my_annotation = HeatmapAnnotation(df = my_ann,

col = list(ImmClust = c("CS1" = mycol[7],"CS2" = mycol[5],"CS3" = mycol[3],"CS4" = mycol[1])))

p <- oncoPrint(onco.input[,rownames(my_ann)], # this is the detailed mutation matrix

alter_fun = alter_fun, # this is the alteration function we already set

col = col, # this is the color for mutation

bottom_annotation = my_annotation, # this is the annotation bar which was put bottom

column_order = rownames(my_ann), # sort your sample as your subtype order

row_order = mut.order, # sort mutation as mutation frequency

show_pct = T, #show percentage in left

column_title = "", # no title shown

show_heatmap_legend=T, # show legend in the oncoprint

column_split = my_ann$ImmClust,

# some detailed size below and you may not have to change it

column_title_gp = gpar(fontsize = 8),

row_names_gp = gpar(fontsize = 8),

column_names_gp = gpar(fontsize = 8))

pdf(file.path(fig.path,"oncoprint of coadread differential mutated genes for ImmClust in tcga.pdf"), width = 10,height = 4)

draw(p)

invisible(dev.off())

## mutational signature APOBEC

library(deconstructSigs)

tmp <- maf

tmp$Tumor_Sample_Barcode <- paste0(tmp$Tumor_Sample_Barcode,"A")

maf2 <- read.maf(tmp[which(tmp$Tumor_Sample_Barcode %in% rownames(annCol)),])

maf.tnm = trinucleotideMatrix(maf = maf2, prefix = 'chr', add = TRUE, ref_genome = "BSgenome.Hsapiens.UCSC.hg19")

APOBEC <- as.data.frame(maf.tnm$APOBEC_scores)

APOBEC$ImmClust <- annCol[APOBEC$Tumor_Sample_Barcode,"ImmClust"]

tmp$Chromosome <- paste0("chr",tmp$Chromosome)

unique(tmp$Variant_Classification)

sigs.input.sbs <- mut.to.sigs.input(mut.ref = as.data.frame(tmp),

sample.id = "Tumor_Sample_Barcode",

chr = "Chromosome",

pos = "Start_Position",

ref = "Reference_Allele",

alt = "Tumor_Seq_Allele2",

bsg = BSgenome.Hsapiens.UCSC.hg19,

sig.type = "SBS")

write.csv(sigs.input.sbs,file.path(res.path,"mutation.sig.input.bydeconstructSigs.csv"),row.names = T,quote = F)

cut.off <- 0

mut.wt.cosmic2013 <- mut.wt.cosmic2019 <- data.frame()

sigs.out.cosmic2013.list <- sigs.out.cosmic2019.list <- list()

for (sample in rownames(sigs.input.sbs)) {

tmp <- whichSignatures(tumor.ref = sigs.input.sbs,

signatures.ref = signatures.cosmic,

sample.id = sample,

contexts.needed = TRUE,

tri.counts.method = 'exome2genome',

signature.cutoff = cut.off)

sigs.out.cosmic2013.list[[sample]] <- tmp

tmp <- data.frame(c(tmp$weights,unknown=tmp$unknown),row.names = sample)

mut.wt.cosmic2013 <- rbind.data.frame(mut.wt.cosmic2013,tmp)

tmp <- whichSignatures(tumor.ref = sigs.input.sbs,

signatures.ref = signatures.exome.cosmic.v3.may2019,

sample.id = sample,

contexts.needed = TRUE,

tri.counts.method = 'exome2genome',

signature.cutoff = cut.off)

sigs.out.cosmic2019.list[[sample]] <- tmp

tmp <- data.frame(c(tmp$weights,unknown=tmp$unknown),row.names = sample)

mut.wt.cosmic2019 <- rbind.data.frame(mut.wt.cosmic2019,tmp)

}

write.csv(mut.wt.cosmic2013,file.path(res.path,"mutation.snp.signature.weightMatrix.cosmic2013.csv"),row.names = T,quote = F)

write.csv(mut.wt.cosmic2019,file.path(res.path,"mutation.snp.signature.weightMatrix.cosmic2019.csv"),row.names = T,quote = F)

mut.wt.cosmic2013.trim <- mut.wt.cosmic2013[,1:30]

mut.wt.cosmic2013.trim <- mut.wt.cosmic2013.trim[,colSums(mut.wt.cosmic2013.trim) > 0]

mut.wt.cosmic2013.trim.backup <- mut.wt.cosmic2013.trim

plotdata <- t(mut.wt.cosmic2013.trim[c(sample.C1,sample.C2,sample.C3,sample.C4),])

plotdata[plotdata < 0.1] <- 0

pheatmap(plotdata,

border_color = NA,

annotation_col = annCol[c(sample.C1,sample.C2,sample.C3,sample.C4),c("ImmClust"),drop = F],

annotation_colors = annColors["ImmClust"],

color = NMF:::ccRamp(x = c("#EAF0FA","#6081C3","#3454A7"),n = 64),

cluster_cols = F,

cluster_rows = F,

show_rownames = T,

show_colnames = F,

cellwidth = 0.8,

cellheight = 10)

mut.wt.cosmic2013$APOBEC <- mut.wt.cosmic2013$Signature.2 + mut.wt.cosmic2013$Signature.13

mut.wt.cosmic2013$ImmClust <- annCol[rownames(mut.wt.cosmic2013),"ImmClust"]

boxplot(mut.wt.cosmic2013$APOBEC~mut.wt.cosmic2013$ImmClust)

kruskal.test(mut.wt.cosmic2013$APOBEC~mut.wt.cosmic2013$ImmClust)

mut.wt.cosmic2013$TIDE <- annCol[rownames(mut.wt.cosmic2013),"TIDE"]

mut.wt.cosmic2013$IES <- annCol[rownames(mut.wt.cosmic2013),"IES"]

mut.wt.cosmic2013$SES <- annCol[rownames(mut.wt.cosmic2013),"SES"]

cor.test(mut.wt.cosmic2013$APOBEC,mut.wt.cosmic2013$TIDE, method = "spearman")

cor.test(mut.wt.cosmic2013$APOBEC,mut.wt.cosmic2013$IES, method = "spearman")

cor.test(mut.wt.cosmic2013$APOBEC,mut.wt.cosmic2013$SES, method = "spearman")

se <- function(x) sqrt(var(x) / length(x))

data_summary <- function(data, varname, groupnames){

require(plyr)

summary_func <- function(x, col){

c(mean = mean(x[[col]], na.rm=TRUE),

se = se(x[[col]]))

}

data_sum<-ddply(data, groupnames, .fun=summary_func,

varname)

data_sum <- rename(data_sum, c("mean" = varname))

return(data_sum)

}

df2 <- data_summary(mut.wt.cosmic2013, varname="APOBEC",

groupnames="ImmClust")

df2 <- na.omit(df2)

df2$ImmClust=as.factor(df2$ImmClust)

head(df2)

p<- ggplot(df2, aes(x=ImmClust, y=APOBEC, fill=ImmClust)) +

geom_bar(stat="identity", color="black",

position=position_dodge()) +

geom_errorbar(aes(ymin=APOBEC-se, ymax=APOBEC+se), width=.2,

position=position_dodge(.9))

print(p)

# Finished bar plot

p + labs(title="", x="ImmClust", y = "APOBEC")+

theme_classic() +

scale_fill_manual(values=mycol[c(7,5,3,1)])

ggsave(filename = file.path(fig.path,"APOBEC mutational signature among ImmClust in tcga kruskal p = 0.039.pdf"), width = 5,height = 5)

tmp <- mut.wt.cosmic2013[,c("APOBEC","IES","SES","TIDE","ImmClust")]

tmp <- na.omit(tmp)

sp <- ggscatter(tmp, x = "APOBEC", y = "TIDE", color = "steelblue", rug = T,

add = "reg.line", # Add regressin line

add.params = list(color = "firebrick1", fill = "lightgray"), # Customize reg. line

conf.int = TRUE # Add confidence interval

)

sp + stat_cor(method = "spearman", label.x = 0.2, label.y = 3)

ggsave(filename = file.path(fig.path,"correlation between TIDE and APOBEC in tcga.pdf"), width = 5,height = 5)

sp <- ggscatter(tmp, x = "APOBEC", y = "IES", color = "steelblue", rug = T,

add = "reg.line", # Add regressin line

add.params = list(color = "firebrick1", fill = "lightgray"), # Customize reg. line

conf.int = TRUE # Add confidence interval

)

sp + stat_cor(method = "spearman", label.x = 0.2, label.y = 2)

ggsave(filename = file.path(fig.path,"correlation between IES and APOBEC in tcga.pdf"), width = 5,height = 5)

sp <- ggscatter(tmp, x = "APOBEC", y = "SES", color = "steelblue", rug = T,

add = "reg.line", # Add regressin line

add.params = list(color = "firebrick1", fill = "lightgray"), # Customize reg. line

conf.int = TRUE # Add confidence interval

)

sp + stat_cor(method = "spearman", label.x = 0.2, label.y = 2)

ggsave(filename = file.path(fig.path,"correlation between SES and APOBEC in tcga.pdf"), width = 5,height = 5)

## FGA

segment <- cnv[,c(1:4,6)]

colnames(segment) <- c("sample","chrom","start","end","value")

coadread.movics <- list("clust.res" = annCol,

"mo.method" = "coadread.icluster")

coadread.movics$clust.res$samID <- rownames(coadread.movics$clust.res)

coadread.movics$clust.res$clust <- sapply(coadread.movics$clust.res$ImmClust,

switch,

"CS1" = 1,

"CS2" = 2,

"CS3" = 3,

"CS4" = 4)

coadread.movics$clust.res <- coadread.movics$clust.res[intersect(rownames(coadread.movics$clust.res),unique(segment$sample)),]

fga.immclust <- compFGA(moic.res = coadread.movics,

segment = segment,

iscopynumber = FALSE, # this is a segmented copy number file

cnathreshold = 0.2, # threshold to determine CNA gain or loss

test.method = "nonparametric", # statistical testing method

fig.path = fig.path,

fig.name = "barplot of fga")

## clinical features

coadread.movics$clust.res$samID <- rownames(coadread.movics$clust.res)

coadread.movics$clust.res$clust <- sapply(coadread.movics$clust.res$ImmClust,

switch,

"CS1" = 1,

"CS2" = 2,

"CS3" = 3,

"CS4" = 4)

tmp <- annCol[,c(2:11,14:18)]

tmp$pStage <- as.character(tmp$pStage)

tmp$tStage <- as.character(tmp$tStage)

tmp$nStage <- as.character(tmp$nStage)

tmp$mStage <- as.character(tmp$mStage)

tmp$lymphatic_invasion <- as.character(tmp$lymphatic_invasion)

tmp$colon_polyps <- as.character(tmp$colon_polyps)

tmp$perineural_invasion <- as.character(tmp$perineural_invasion)

tmp$primary_site <- as.character(tmp$primary_site)

tmp[tmp == "Missing"] <- NA

clin.icluster <- MOVICS::compClinvar(moic.res = coadread.movics,

includeNA = FALSE,

res.path = res.path,

var2comp = tmp, # data.frame needs to summarize (must has row names of samples)

strata = "ImmClust", # stratifying variable (e.g., Subtype in this example)

factorVars = c("Age","Sex","pStage","tStage","nStage","mStage","lymphatic_invasion","colon_polyps","perineural_invasion","primary_site","Responder"), # features that are considered categorical variables

nonnormalVars = c("TIDE","IES","SES"), # feature(s) that are considered using nonparametric test

exactVars = c("Age","Sex","pStage","tStage","nStage","mStage","lymphatic_invasion","colon_polyps","perineural_invasion","primary_site","Responder"), # feature(s) that are considered using exact test

doWord = TRUE, # generate .docx file in local path

tab.name = "summarization of clinical features between immclust in tcga coadread")

#---------------------------------------#

# difference of immune-related features #

### TCGA

## neoantigen

neoantigen <- read.table(file.path(data.path,"Predicted Neoantigen.txt"),sep = "\t",row.names = NULL,check.names = F,stringsAsFactors = F,header = T)

neoantigen$samID <- substr(neoantigen$PatientID,9,12)

immclust <- annCol

immclust$samID <- substr(rownames(immclust),9,12)

comID <- intersect(neoantigen$samID, immclust$samID)

neoantigen <- neoantigen[which(neoantigen$samID %in% comID),]

matchID <- match(neoantigen$samID,immclust$samID)

immclust[matchID,"PredNeo"] <- neoantigen$`Predicted NeoAgs`

tmp <- data.frame(Neo = immclust$PredNeo,

ImmClust = immclust$ImmClust)

tmp <- as.data.frame(na.omit(tmp))

my_comparisons <- list( c("CS1", "CS2"),

c("CS1", "CS3"),

c("CS1", "CS4"))

ggplot(data = tmp,aes(x = ImmClust,

y = log2(Neo + 1),

fill = ImmClust))+

scale_fill_manual(values = mycol[c(7,5,3,1)]) +

geom_violin(alpha = 0.4, position = position_dodge(width = .75),

size = 0.8, color="black") +

geom_boxplot(notch = FALSE, outlier.size = -1,

color="black", lwd=0.8, alpha = 0.7) +

geom_point(shape = 21, size=2,

position = position_jitterdodge(),

color="black", alpha = 1) +

theme_bw() +

ylab("log2(predicted neoantigen)") +

xlab("") +

theme(axis.text.x = element_text(hjust = 1, size = 12, color = "black"),

axis.ticks = element_line(size=0.2, color="black"),

axis.ticks.length = unit(0.2, "cm"),

legend.position = "top",

panel.background = element_blank(),

panel.grid = element_blank(),

axis.title = element_text(size = 12),

axis.text = element_text(size = 12)) +

stat_compare_means(comparisons = my_comparisons,method = "wilcox.test") +

stat_compare_means(method = "kruskal.test", label.y = min(log2(tmp$Neo + 1))-0.5)

ggsave(file.path(fig.path,"boxplot for predicted neoantigen in immclust of tcga coadread.pdf"),width = 4,height = 5)

## immune score

tmp <- data.frame(IES = as.numeric(est.tcga.backup[2,rownames(annCol)]),

ImmClust = annCol$ImmClust)

my_comparisons <- list( c("CS1", "CS2"),

c("CS1", "CS3"),

c("CS1", "CS4"))

ggplot(data = tmp,aes(x = ImmClust,

y = IES,

fill = ImmClust))+

scale_fill_manual(values = mycol[c(7,5,3,1)]) +

geom_violin(alpha = 0.4, position = position_dodge(width = .75),

size = 0.8, color="black") +

geom_boxplot(notch = TRUE, outlier.size = -1,

color="black", lwd=0.8, alpha = 0.7) +

geom_point(shape = 21, size=2,

position = position_jitterdodge(),

color="black", alpha = 1) +

theme_bw() +

ylab("Immune enrichment score") +

xlab("") +

theme(axis.text.x = element_text(hjust = 1, size = 12, color = "black"),

axis.ticks = element_line(size=0.2, color="black"),

axis.ticks.length = unit(0.2, "cm"),

legend.position = "top",

panel.background = element_blank(),

panel.grid = element_blank(),

axis.title = element_text(size = 12),

axis.text = element_text(size = 12)) +

stat_compare_means(comparisons = my_comparisons,method = "wilcox.test") +

stat_compare_means(method = "kruskal.test", label.y = -800)

ggsave(file.path(fig.path,"boxplot for predicted IES in immclust of tcga coadread.pdf"),width = 4,height = 5)

## stromal score

tmp <- data.frame(SES = as.numeric(est.tcga.backup[1,rownames(annCol)]),

ImmClust = annCol$ImmClust)

my_comparisons <- list( c("CS1", "CS2"),

c("CS1", "CS3"),

c("CS1", "CS4"))

ggplot(data = tmp,aes(x = ImmClust,

y = SES,

fill = ImmClust))+

scale_fill_manual(values = mycol[c(7,5,3,1)]) +

geom_violin(alpha = 0.4, position = position_dodge(width = .75),

size = 0.8, color="black") +

geom_boxplot(notch = TRUE, outlier.size = -1,

color="black", lwd=0.8, alpha = 0.7) +

geom_point(shape = 21, size=2,

position = position_jitterdodge(),

color="black", alpha = 1) +

theme_bw() +

ylab("Stromal enrichment score") +

xlab("") +

theme(axis.text.x = element_text(hjust = 1, size = 12, color = "black"),

axis.ticks = element_line(size=0.2, color="black"),

axis.ticks.length = unit(0.2, "cm"),

legend.position = "top",

panel.background = element_blank(),

panel.grid = element_blank(),

axis.title = element_text(size = 12),

axis.text = element_text(size = 12)) +

stat_compare_means(comparisons = my_comparisons,method = "wilcox.test") +

stat_compare_means(method = "kruskal.test", label.y = -2000)

ggsave(file.path(fig.path,"boxplot for predicted SES in immclust of tcga coadread.pdf"),width = 4,height = 5)

# tide score

tmp <- data.frame(TIDE = tide.res[rownames(annCol),"TIDE"],

ImmClust = annCol$ImmClust)

my_comparisons <- list( c("CS1", "CS2"),

c("CS1", "CS3"),

c("CS1", "CS4"))

ggplot(data = tmp,aes(x = ImmClust,

y = TIDE,

fill = ImmClust))+

scale_fill_manual(values = mycol[c(7,5,3,1)]) +

geom_violin(alpha = 0.4, position = position_dodge(width = .75),

size = 0.8, color="black") +

geom_boxplot(notch = TRUE, outlier.size = -1,

color="black", lwd=0.8, alpha = 0.7) +

geom_point(shape = 21, size=2,

position = position_jitterdodge(),

color="black", alpha = 1) +

theme_bw() +

ylab("TIDE score") +

xlab("") +

theme(axis.text.x = element_text(hjust = 1, size = 12, color = "black"),

axis.ticks = element_line(size=0.2, color="black"),

axis.ticks.length = unit(0.2, "cm"),

legend.position = "top",

panel.background = element_blank(),

panel.grid = element_blank(),

axis.title = element_text(size = 12),

axis.text = element_text(size = 12)) +

stat_compare_means(comparisons = my_comparisons,method = "wilcox.test") +

stat_compare_means(method = "kruskal.test", label.y = -2)

ggsave(file.path(fig.path,"boxplot for predicted TIDE in immclust of tcga coadread.pdf"),width = 4,height = 5)

#---------------------------------------------#

# calculate MeTIL score using DNA methylation #

orgmeth <- readRDS(file = file.path(data.path,"orgmeth.combat.filtered.rds"))

comsamMeth <- intersect(colnames(orgmeth),rownames(annCol))

MeTIL.marker <- c("cg20792833","cg20425130","cg23642747","cg12069309","cg21554552")

meth.metil <- orgmeth[MeTIL.marker,comsamMeth]

MeTIL <- t(scale(t(meth.metil)))

pca.MeTIL <- prcomp(MeTIL,center = F,scale. = F)

MeTIL <- pca.MeTIL$rotation[,1]

tmp <- data.frame(MeTIL = MeTIL,

ImmClust = annCol[comsamMeth,"ImmClust"])

my_comparisons <- list( c("CS1", "CS2"),

c("CS1", "CS3"),

c("CS1", "CS4"))

ggplot(data = tmp,aes(x = ImmClust,

y = MeTIL,

fill = ImmClust))+

scale_fill_manual(values = mycol[c(7,5,3,1)]) +

geom_violin(alpha = 0.4, position = position_dodge(width = .75),

size = 0.8, color="black") +

geom_boxplot(notch = TRUE, outlier.size = -1,

color="black", lwd=0.8, alpha = 0.7) +

geom_point(shape = 21, size=2,

position = position_jitterdodge(),

color="black", alpha = 1) +

theme_bw() +

ylab("MeTIL score") +

xlab("") +

theme(axis.text.x = element_text(hjust = 1, size = 12, color = "black"),

axis.ticks = element_line(size=0.2, color="black"),

axis.ticks.length = unit(0.2, "cm"),

legend.position = "top",

panel.background = element_blank(),

panel.grid = element_blank(),

axis.title = element_text(size = 12),

axis.text = element_text(size = 12)) +

stat_compare_means(comparisons = my_comparisons,method = "wilcox.test") +

stat_compare_means(method = "kruskal.test", label.y = -0.1)

ggsave(file.path(fig.path,"boxplot for MeTIL in immclust of tcga coadread.pdf"),width = 4,height = 5)

# barplot of tide response

bar.input <- as.data.frame.array(table(annCol$ImmClust,annCol$Responder)[,1:2]); tmp <- dimnames(bar.input)

bar.input <- t(bar.input/rowSums(bar.input))

pdf(file.path(fig.path,"barplot of response in tcga coadread.pdf"), width = 4,height = 5)

par(bty="o", mgp = c(1.5,.33,0), mar=c(5,3,1,1), las=1, tcl=-.25,las = 1, xpd = T)

a <- barplot(as.matrix(bar.input),

border = "black",

names.arg = c("CS1","CS2","CS3","CS4"),

ylab = "Percentage",

col = c("grey90",alpha(purple,0.8)))

text(a[1],bar.input[1,1]/2,round(bar.input[1,1],2)*100, cex = 1)

text(a[1],bar.input[1,1] + (bar.input[2,1])/2,round(bar.input[2,1],2)*100, cex = 1)

text(a[2],bar.input[1,2]/2,round(bar.input[1,2],2)*100, cex = 1)

text(a[2],bar.input[1,2] + (bar.input[2,2])/2,round(bar.input[2,2],2)*100, cex = 1)

text(a[3],bar.input[1,3]/2,round(bar.input[1,3],2)*100, cex = 1)

#text(a[3],bar.input[1,3] + (bar.input[2,3])/2,round(bar.input[2,3],2)*100, cex = 1)

text(a[4],bar.input[1,4]/2,round(bar.input[1,4],2)*100, cex = 1)

text(a[4],bar.input[1,4] + (bar.input[2,4])/2,round(bar.input[2,4],2)*100, cex = 1)

legend(1.5,-0.06, legend = c("Responder","Non-responder"), border = NA, fill = c(alpha(purple,0.8),"grey90"), bty = "n", x.intersp = 0.2, y.intersp = 0.8)

invisible(dev.off())
